# Lifetime physical intimate partner violence (pIPV) among Mozambican women: Individual and contextual level factors driving its prevalence

**DOI:** 10.1371/journal.pone.0312640

**Published:** 2025-12-15

**Authors:** Maud Zinayele Muosieyiri, Nazeem Muhajarine

**Affiliations:** 1 Department of Community Health and Epidemiology, College of Medicine, University of Saskatchewan, Saskatoon, Saskatchewan, Canada; 2 Mozambique-Canada Maternal Health Project, Inhambane, Mozambique, Canada; Utah State University, UNITED STATES OF AMERICA

## Abstract

**Background:**

Intimate partner violence (IPV) remains a significant public health issue in Mozambique. This study uses data from the 2022–2023 Mozambique Demographic and Health Survey (DHS) to examine the prevalence and sociodemographic determinants of Lifetime IPV among women. Specifically, this study focuses on physical IPV (pIPV), as measured in the Mozambique Demographic and Health Survey (DHS), which does not capture sexual or emotional violence.

**Methods:**

A nationally representative sample of 4,813 women aged 15–49 was analyzed to assess the prevalence of Lifetime pIPV. Lifetime pIPV was defined as physical violence perpetrated by a current or former partner, by DHS definitions. Logistic regression models were used to identify individual- and contextual-level factors associated with Lifetime IPV.

**Results:**

Nearly 1 in 4 women (21.92%) reported experiencing physical abuse from a current or former partner in their lifetime. Marital status emerged as a key individual-level determinant, with cohabitating and separated women being at significantly higher odds of experiencing pIPV compared to women who had never been in a union. Educational attainment and current employment were also associated with increased odds of pIPV. Similarly, women who justified physical abuse had higher odds of experiencing pIPV. Additionally, husbands/partners’ alcohol consumption was one of the strongest predictors, nearly tripling the odds of Lifetime pIPV. Finally, the effect modification between marital status and education showed that the effect of marital status on pIPV was further modified by women educational attainment. At the contextual level, provincial disparities were observed, with Cabo Delgado and Manica showing the highest pIPV prevalence, while Inhambane and Gaza had the lowest.

**Conclusion:**

This study provides updated data on the prevalence of Lifetime pIPV in Mozambique and highlights key individual and contextual factors contributing to pIPV. The findings underscore the need for targeted interventions addressing socio-cultural norms, improving educational opportunities, mitigating male partner’s alcohol consumption, and implementing province-specific strategies to reduce pIPV and enhance women’s safety across Mozambique.

## Background

Intimate Partner Violence (IPV) remains a global public health, social, and moral issue, significantly impacting women’s well-being. Addressing IPV is crucial for achieving Sustainable Development Goals (SDG 5: Gender Equality, SDG 4: Quality Education, and SDG 3: Health and Well-being). According to the World Health Organization (WHO), nearly 27% of women aged 15–49 have experienced physical and/or sexual violence from an intimate partner [1]. IPV results in severe health consequences, including injuries, mental health disorders, chronic conditions, and pregnancy complications such as miscarriages and preterm births [1–3]. While IPV affects all societies, its prevalence is highest in Latin America (31%), Southeast Asia (33%), and Sub-Saharan Africa (33%) [1,4].

In Sub-Saharan Africa, IPV prevalence differs by geographic location, likely due to variations in socio-cultural beliefs, gender norms, and economic inequality. For instance, a multi-country study in SSA showed that 50% of women in Ethiopia reported having experienced IPV in their lifetime, while only 17% of women in Namibia had ever experienced IPV [5,6]. Another pooled analysis of 26 African countries found that while 14% of pregnant women in South Africa having experienced physical violence during pregnancy, only 2.1% of those in Burkina Faso reported experiencing the same [7]. Ahinkorah and colleagues determined that Gabon had the highest prevalence of IPV against women (45.3%), while Comoros had the lowest prevalence (4.9%) in their study of 84,486 women across 18 countries [3]. It is clear, given these reported data from across numerous countries, that understanding and addressing the specific socio-cultural, economic and gendered factors driving IPV at individual and contextual levels is essential for creating safer societies for women across Africa [4,8].

African cultural traditions and gender norms widely reinforce men’s dominance as breadwinners and decision-makers in intimate partner relationships, making women more vulnerable to unjust treatment [9,10]. These norms intersect and interact with individual characteristics such as marital status, education, employment, attitudes towards abuse, and substance use; this shapes women’s susceptibility to, or protection from, IPV. The prevailing literature demonstrates that cohabitating/married women have a higher likelihood of experiencing IPV than their single counterparts. Cruz et al. revealed that cohabitating/married women had 1.53 times higher odds of experiencing physical violence compared to single women (OR = 2.53, 95% CI = 1.22, 4.74) [6]. Ahinkorah and colleagues, in a notable finding, reported that cohabitating women are more likely to experience IPV than married women; possibly because women often may compromise more once they are married or feel that they have fewer options, resulting in less conflict with their husbands [3].

Evidence also suggests that a woman’s higher level of education and employment, both general markers of women empowerment, can either reduce susceptibility to or trigger IPV, depending on the context [11,12]. On one hand, some studies show that higher education level and employment reduce the odds of IPV by increasing social networks and support, sources of information, leading to greater autonomy and better bargaining power in relationships [3,4,8,13]. For example, a study analyzing IPV among women across 16 Indian states determined that women with a higher than secondary education were 59% less likely to experience IPV compared to women with no formal education (OR = 0.41, 95% CI = 0.36, 0.46).(13) In contrast, in communities with rigid gender roles and acceptable views of and relaxed attitudes about physical violence, women’s higher education and employment increase their risk of experiencing IPV [8]. Cools and Kotsadam revealed that African women who achieved either a primary or a secondary education were significantly more likely to experience IPV, compared to those without formal education, the likelihood rising by 5.3 and 3.1 percentage points, respectively. Ahinkorah and colleagues (2018) showed that employed women had 33% higher odds of experiencing physical violence from their husbands/partners (OR = 1.33, 95% CI = 1.28, 1.37) compared to non-employed women [4,7].

Researchers have hypothesized that women’s empowerment and IPV occur because empowered women challenge traditional, unfavorable (to women) gender roles, including questioning male authority [8,14]. Additionally, women’s attitudes towards IPV shape their vulnerability to experiencing it [15]. A population-based survey showed that IPV justification trends align with global IPV prevalence, with higher justification rates in South and Southeast Asia (47%) and Sub-Saharan Africa (38%) compared to Central and West Asia and Europe (29%) [15,16]. Another study of African countries showed that pregnant women who justified IPV had a higher likelihood of experiencing physical violence than those who did not [7]. Scientists argue that in societies where women justify IPV, they are less likely to oppose it or to report it, thus increasing its occurrence [17]. Furthermore, alcohol use and abuse by women’s husbands/partners are consistently linked to higher rates of IPV [18]. Alcohol consumption can lower inhibitions, impair functioning, and heighten depressive symptoms, all of which may increase the likelihood of violence against women [7].

At the contextual level, access to resource and wealth (as measured by wealth index), and rural/urban living have been reported to influence IPV trends. The Wealth Index is an aggregate score that measures the relative wealth of household’s wealth and may serve as a proxy for socioeconomic status [3,19]. Generally, women in the poorest wealth status are more likely to experience IPV than those in the richest category [3,8]. Stockl et al. showed that there is a significant decrease in the odds of experiencing IPV in richer households, compared to those in the middle and poorest tertile of the wealth measurement [8]. A strong supporting argument is that women within the richest wealth group are more resourced to fight for their rights and seek help against physical abuse compared to those of the poorest index [7]. Another argument is that financial stress is likely to be the lesser reason for conflict in these well-endowed households [3,8]. There is mixed evidence concerning the influence of rural/urban living and IPV prevalence. Some studies show that rural living increases the odds of IPV due to rigid gender norms [6], while others suggest that living in rural settings decreases IPV risk since women in these settings maybe more subservient, thus reducing any resistance to their husbands/partners dominance and aggression [7,17,20].

Mozambique, a southeastern African country, has historically had one of the continent’s highest IPV prevalence; the 2011 DHS report indicated that 33% of women had experienced physical violence since age 15 [17]. Nevertheless, research suggests that this statistic is lower than the actual estimate due to underreporting, since IPV is often seen as a private issue, discouraging women from reporting violence and seeking support [6,17,20,21]. For instance, a 2011 study conducted in Zambezia, a central province in Mozambique, portrayed that 70% of participants admitted they never sought help or disclosed incidents of violence against them [17]. Despite historical reports, current data on IPV and its structural and sociodemographic drivers are largely unknown in Mozambique. This data gap makes it challenging to develop and implement effective policies and protocols to ensure the welfare and safety of women across the country. Nationally, the Mozambique Constitution establishes gender equality in all areas of society and prohibits all legislative, political, cultural, economic, and social discrimination. Many state bodies tasked with preventing and ending gender-based violence exists. However, lack of reliable and country-wide contemporary data hampers evidence-based action on this front. This present study, therefore, sought to address this evidence gap by utilizing the most recent Mozambique DHS data (2022–2023) to investigate the Lifetime physical IPV prevalence and its associated sociodemographic and structural factors among 15- to 49-year-old women in Mozambique. While IPV encompasses physical, sexual, and emotional violence, this study focuses exclusively on physical IPV (pIPV), following the DHS definition. This study focuses on pIPV consistent with the systematic measurement in DHS surveys, ensuring data reliability and comparability across studies. Physical IPV is also the most widely recognized in legal and policy frameworks, making it a critical focus for intervention. Additionally, many DHS-based studies in sub-Saharan Africa prioritize physical IPV, allowing for meaningful regional and global comparisons.

## Methods

### Data source

The present study analyzes information from a secondary data source, the 2022–2023 Mozambique DHS data [19]. The DHS is a global survey that is conducted in over 85 low- and middle-income countries. We received approval from the DHS program to access de-identified datasets for the 2022–2023 Mozambique reports, which were provided on June 17, 2024. Thus, participant confidentiality was maintained throughout our analyses, and no information can be directly linked to any individual.

The 2022−23 Mozambique DHS was a nationwide, population-based cross-sectional survey that covered all 10 provinces (Niassa, Cabo Delgado, Nampula, Zambezia, Tete, Manica, Sofala, Inhambane, Gaza, and Maputo) as well as the capital city region of Maputo, which holds provincial status. Data collection followed a two-stage stratified sampling design. In the first stage, clusters (enumeration areas, EAs) were selected based on “IV Recenseamento Geral da População e Habitação 2017” (IV RGPH 2017)) [22]. A total of 619 recorded areas were chosen using probability-proportional-to-size, determined by the number of households in each explicit stratum. In the second stage, 26 households were systematically selected equally from each area. This process resulted in the selection of 16,045 households for data collection. All women aged 15–49 years who were either residents or visitors in the household the night before the interviews were eligible to participate. In a subsample of half of the selected households, all men aged 15–54 years were also eligible for interviews [19]. For our analyses, we utilized data from a sub-group of women within the Individual Women Recode (IR) file who were randomly selected to complete the Domestic Violence module (N = 4,813).

### Variables

#### Outcome variable.

We define the outcome variable, Lifetime Physical Intimate Partner Violence (pIPV), as any physical violence experienced by women from a current or former partner since the age of 15 as defined by the DHS. The DHS criteria for pIPV is defined as those women who were ever-married aged 15–49 and those who were never-married aged 15–49 who have ever had an intimate partner and have experienced physical violence by their current or most recent husband/intimate partner [23]. A “Yes” response to any of the following questions meets the criteria for Lifetime pIPV (a) Ever been pushed, shook or had something thrown by husband/partner; (b) Ever been slapped by husband/partner (c) Ever been punched with fist or hit by something harmful by husband/partner (d) Ever been kicked or dragged by husband/partner (e) Ever been strangled or burnt by husband/partner (f) Ever been attacked with knife/gun or other weapon by husband/partner (g) Ever CS physical violence by husband/partner (h) Ever had arm twisted or hair pulled by husband/partner (i) Previous husband: ever hit, slap, kick or physically hurt respondent. The outcome variable was dichotomized (“Yes”/ “No”) for all analyses.

#### Independent variables.

Seminal articles on IPV in Sub-Saharan Africa, like that of Ahinkorah et [7] as well as the adapted theoretical framework from Azevêdo et al [24] (**S1 Fig**), guided the selection of the independent variables. These variables were divided into Individual-level and Contextual-level factors. The Individual-level factors include (a) Maternal Age (b) Husband/Partner’s Age (c) Maternal Educational Level (d) Husband/Partner’s Educational Level (e) Woman’s Marital Status (f) Woman’s Current Employment Status (g) Woman’s Access to Media (h) Woman’s Justification for Beatings and (i) Husband/Partner’s Alcohol Intake (j) Age Gap between Respondent and Husband/Partner (k) Woman’s Religion (l) Sex of Household Head (m) Household Size (n) Polygamy. Contextual-level variables, but applied at individual level, comprised of (a) Wealth Index (b) Type of Place of Residence, and (c) Province of Residence. The supplemental material contains all the details of these variables, including their categorizations (**S2 Table**).

### Data analysis

**Fig 1:**
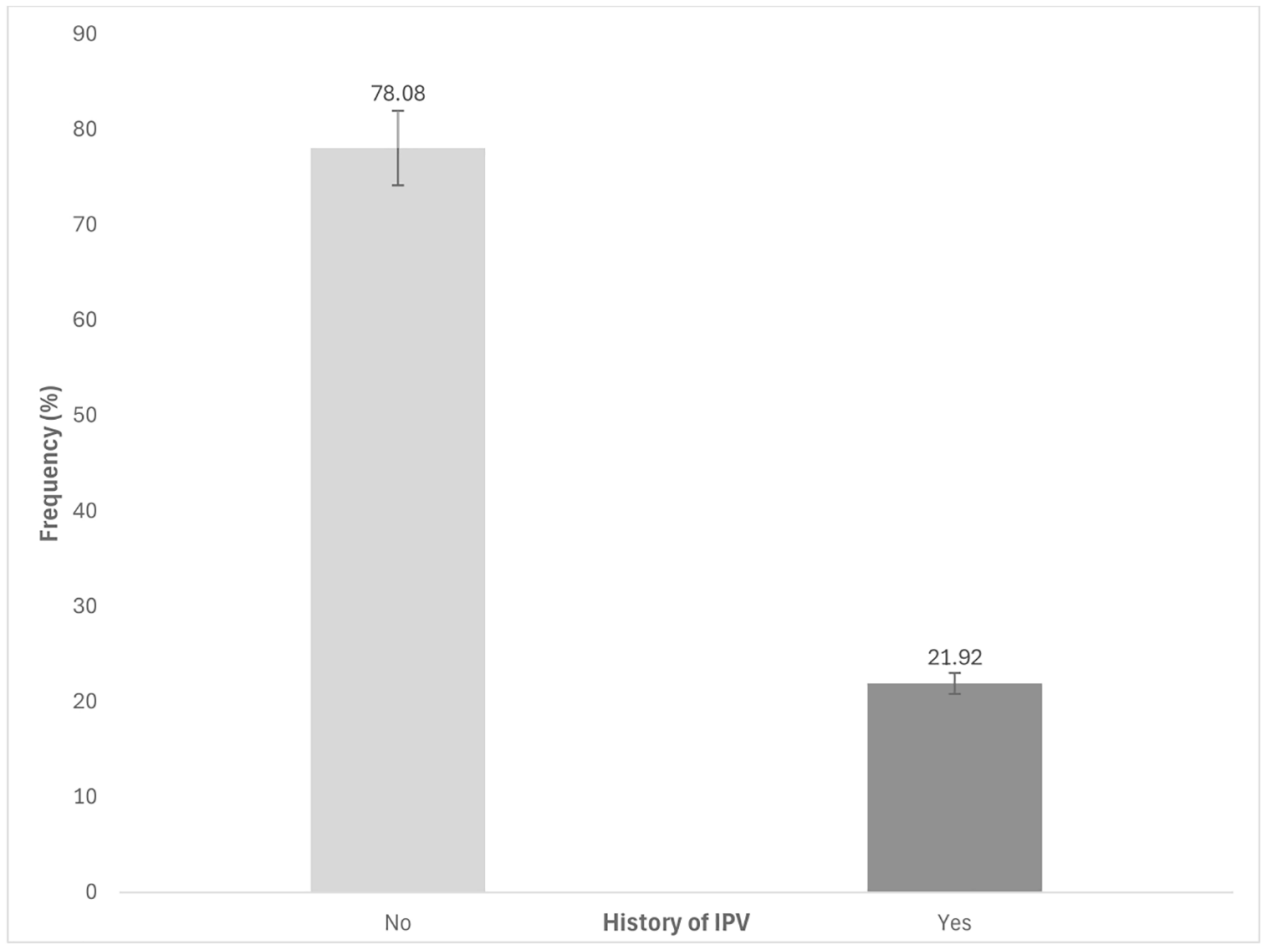
The prevalence of lifetime intimate partner violence (pIPV) among women in Mozambique, based on the 2022-2023 DHS study (N = 4813). The Lifetime prevalence of pIPV was 21.92% across the population.

Study population characteristics were summarized as counts and frequencies for all independent variables and presented in Table 1. Lifetime pIPV (**Fig 1**) as well as pIPV experienced within the last 12 months (**S3 Fig**) were also quantified as counts and frequencies and presented as figures. Bivariate analyses were conducted using chi-square tests to estimate unadjusted odds ratios (ORs), while multivariable logistic regression models were used to compute adjusted odds ratios (aORs) (**S5 Table**). In the bivariate analysis, variables with a p-value ≤ 0.20 were considered for inclusion in the multivariable logistic regression model. This threshold is commonly used in epidemiologic research to avoid excluding potentially important predictors that may become significant after adjusting for confounding factors. The final model was refined through a stepwise selection process, retaining significant (p < 0.05) or theoretically relevant variables based on prior literature and the study’s conceptual framework. Selected (based on theoretical framework) variables were also tested for effect modifications. Finally, the Hosmer-Lemeshow test was used to determine the model’s goodness-of-fit. Because of smaller cell counts within the highest 2 categories of the variables: “Husband/Partner’s Age” and “Husband/Partner’s Educational Level”, these categories were merged to ensure sufficient sample in both the bivariate and multivariate analyses. All adjusted odds ratios, their 95% CIs, and corresponding p-values are reported in Table 2. The analyses were performed using SAS 9.4M8.all proportions, odds ratios, and statistical inferences are based on weighted data, following DHS analytical guidelines.

**Table 1:** Background information on study population (N = 4,813) from demographic and health survey, 2022–2023, Mozambique.

Variable	Frequency (N)	Frequency (%)
**Woman’s Age (N = 4813)**		
15–24	2002.47	41.61
25–34	1379.76	28.67
35–44	1020.49	21.20
≥ 45	410.28	8.52
**Husband/Partner’s Age (N = 3143)**		
15–24	426.79	13.58
25–34	1066.48	33.93
35–44	860.18	27.37
≥ 45	789.66	25.12
**Woman’s Marital Status (N = 4813)**		
Never in Union	991.37	20.60
Married	1328.18	27.60
Living with a Partner	1814.93	37.71
No longer living together/separated	678.52	14.10
**Woman’s Educational Level (N = 4813)**		
No Formal Education	1318.52	27.40
Primary	2052.57	42.65
Secondary	1308.88	27.19
Higher	133.03	2.76
**Husband/Partner’s Educational Level (N = 3143)**		
No Formal Education	1088.17	34.62
Primary	1190.33	37.87
Secondary	755.65	24.04
Higher	108.96	3.47
**Woman’s Current Employment Status (N = 4813)**		
No	3368.94	70.00
Yes	1444.06	30.00
**Woman’s Access to Media (N = 4813)**		
Less than Once a Week	3014.49	62.63
At Least Once a Week	1798.51	37.37
**Woman’s Justification for Beating from Husband/Partner (N = 4813)**		
No justification	3904.05	81.11
Moderate Justification	471.29	9.79
Moderate-to-complete Justification	437.66	9.09
**Husband/Partner’s Alcohol Consumption (N = 4323)**		
No	3102.21	71.76
Yes	1221.02	28.24
**Age Gap between Respondent and Husband/Partner (N = 4813)**		
Husband’s Younger	1857.15	38.59
No Age Difference	105.96	2.20
Husband 1–4 Years Older	1114.59	23.16
Husband 5–9 Years Older	1029.66	21.39
Husband 10 + Years Older	705.65	14.66
**Woman’s Religion (N = 4813)**		
No Specified Religion	390.27	8.11
Catholic	1447.89	30.08
Other Christian	1968.45	40.90
Muslim	1006.40	20.91
**Sex of Household Head (N = 4813)**		
Male	3344.68	69.49
Female	1468.33	30.51
**House Hold Size (N = 4813)**		
Single Member	56.48	1.17
2–3 Members	100.68	2.09
4–5 Members	88.58	1.84
6–7 Members	114.77	2.38
8 + Members	4452.48	92.51
**Polygamy (N = 3044)**		
No Other Wives	2583.89	84.88
1 Other Wife	374.71	12.31
2 + Other Wives	85.09	2.80
**Husband/Partner’s Current Employment Status (N = 3039)**		
No	926.26	30.48
Yes	2112.58	69.52
**Wealth Index (N = 4813)**		
Poorest	927.58	19.27
Poorer	951.07	19.76
Middle	905.31	18.81
Richer	968.80	20.13
Richest	1060.25	22.03
**Type of Place of Residence (N = 4813)**		
Rural	2961.09	61.52
Urban	1851.91	38.48
**Province of Residence (N = 4813)**		
Niassa	332.47	6.91
Cabo Delgado	263.64	5.48
Nampula	1165.93	24.22
Zambézia	728.15	15.13
Tete	494.14	10.27
Manica	323.01	6.71
Sofala	340.92	7.08
Inhambane	206.70	4.29
Gaza	250.91	5.21
Maputo	474.46	9.86
Cidade de Maputo	232.67	4.83

*The total study population consists of 4,813 women; however, the number of observations varies across variables due to differences in eligibility criteria within the DHS dataset. For instance, variables related to husbands/partners apply only to women who reported having a partner, resulting in a smaller denominator. The sample size (N) for each variable is specified accordingly.

¶ Weighted frequencies derived using DHS survey weights. Percentages may not sum to 100% due to rounding

ªWealth Index: Composite score derived from participants’ household assets using principal component analysis

**Table 2:** Results from the multivariable analysis, full model showing significant factors at individual- and context-level and lifetime physical intimate partner violence (pIPV) from demographic and health survey, 2022–2023, Mozambique.

	Lifetime pIPV	
Independent Variables	Adjusted OR [95% CI]	p-value
**Woman’s Age**		0.3871
15–24	1.00 [Ref]	[Ref]
25–34	1.101 [0.890,1.362]	0.1264
35–44	1.039 [0.801,1.347]	0.5853
≥ 45	0.867 [0.622,1.207]	0.1785
**Husband/Partner’s Age**		0.1029
15–24	1.00 [Ref]	[Ref]
25–34	1.250 [0.922,1.694]	0.6648
≥ 35	1.426 [1.025,1.985]	0.0402
**Woman’s Marital Status**		**<0.0001**
Never in Union	1.00 [Ref]	[Ref]
Married	0.972 [0.747, 1.264]	0.8345
Living with a Partner	1.617 [1.293, 2.022]	<0.0001
No longer living together/separated	1.787 [1.377, 2.320]	<0.0001
**Woman’s Educational Level**		0.0894
No Formal Education	1.00 [Ref]	[Ref]
Primary	1.044 [0.855, 1.273]	0.6808
Secondary	0.896 [0.733, 1.096]	0.2562
Higher	0.656[0.455, 0.945]	0.0292
**Husband/Partner’s Educational Level**		0.3465
No Formal Education	1.00 [Ref]	[Ref]
Primary	1.044 [0.844,1.291]	0.5841
Secondary Education or Higher	0.876 [0.671,1.145]	0.3878
**Woman’s Current Employment Status**		**0.0004**
No	1.00 [Ref]	[Ref]
Yes	1.339 [1.138,1.576]	0.0004
**Woman’s Justification for Beating**		0.0059
No justification	1.00 [Ref]	[Ref]
Moderate Justification	1.408 [1.116,1.776]	0.0963
Moderate-to-complete Justification	1.286 [0.971,1.703]	0.5865
**Husband/Partner’s Alcohol Consumption**		**<0.0001**
No	1.00 [Ref]	[Ref]
Yes	2.928 [2.491,3.441]	<0.0001
**Type of Place of Residence**		0.1515
Rural	0.877 [0.734,1.049]	[Ref]
Urban	1.00 [Ref]	0.1515
**Province of Residence**		**<0.0001**
Niassa	0.445 [0.293,0.676]	<0.0001
Cabo Delgado	1.640 [1.162,2.315]	<0.0001
Nampula	1.340 [0.939,1.914]	0.0266
Zambézia	1.034 [0.708,1.510]	0.9791
Tete	1.139 [0.807,1.607]	0.4186
Manica	2.660 [1.897,3.732]	<0.0001
Sofala	1.143 [0.822,1.589]	0.3919
Inhambane	0.679 [0.473,0.974]	0.0017
Gaza	0.570 [0.393,0.828]	<0.0001
Maputo	1.00 [Ref]	[Ref]
Cidade de Maputo	1.108 [0.786,1.562]	0.6279
**Woman’s Educational Level X Woman’s Marital Status**		**0.0078**
Primary X Married	0.855[0.620,1.178]	0.0656
Primary X Living with a Partner	1.554[1.180, 2.048]	0.0263
Primary X No longer living together/separated	0.911[0.594, 1.398]	0.7626
Secondary X Married	0.865[0.555, 1.346]	0.1583
Secondary X Living with a Partner	1.017[0.714, 1.447]	0.1339
Secondary X No longer living together/separated	0.978[0.596, 1.607]	0.7910
Higher X Married	0.195[0.065, 0.584]	0.0329
Higher X Living with a Partner	0.958[0.417, 2.201]	0.3288
Higher X No longer living together/separated	0.791[0.264, 2.366]	0.4183
**Model Fitness**	**Chi-Square**	**p-value**
Hosmer-Lemeshow	4.7514	0.7838

^**κ**^All boldened p-values are statistically significant

^**φ**^Interaction Term: Woman’s Education X Marital Status

ªFull list of variables included in initial analysis: Woman’s Age, Husband/Partner’s Age, Woman’s Marital Status, Woman’s Educational Level, Husband/Partner’s Educational Level, Woman’s Current Employment Status, Woman’s Access to Media, Woman’s Justification for Beating from Husband, Husband/Partner’s Alcohol Consumption, Wealth Index, Type of Place of Residence, Province of Residence. All non-significant variables (p < 0.20) were subsequently excluded from multivariate analysis.

## Results

### Sample characteristics

Table 1 summarizes the background characteristics of the study population. The study population consisted of 4,813 women in Mozambique, with the largest proportion being between the ages of 15 and 24 (41.61%). In contrast, the majority of their husbands/partners were in the 25–34 age range (33.93%). Regarding marital status, most participants were either living with a partner (37.71%) or were married (27.60%). Educationally, the largest proportion of women (42.65%), and their husbands/partners (37.87%), had completed primary education, while only 2.76% and 3.47% had attained an education higher than secondary level, respectively. Concerning employment status, 70.00% of the women were unemployed at the time of the survey. Contrastingly, 69.52% of husbands/partners were employed, while 30.48% were not working. Media access was limited, with more than half of the women reporting less than once-weekly access (62.63%). In terms of stating pIPV was justified, the vast majority of women did not justify violence (81.11%). Almost one-third of the women stated that their husbands/partners (28.24%) consumed alcohol. Additional household and demographic characteristics were examined. The age gap between respondents and their partners varied, with 23.16% of women having a husband/partner who was 1–4 years older, while 14.66% had a partner 10 + years older. Polygamous unions were present among 15.11% of the women, with 2.80% having at least two co-wives. The sex of the household head was male in 69.49% of households. Regarding household size, 1.84% of women resided in households with 4–5 members, while 92.51% lived in households with 8 or more members.

On a contextual level, Rural living was prevalent among the participants, with 61.52% residing in rural areas. The wealth distribution showed an even spread, with 19.27% in the poorest category and 22.03% in the richest category. Of the participants, 4.29% resided in Inhambane while 24.22% lived in Nampula province.

### Prevalence of lifetime pIPV

**Fig 2:**
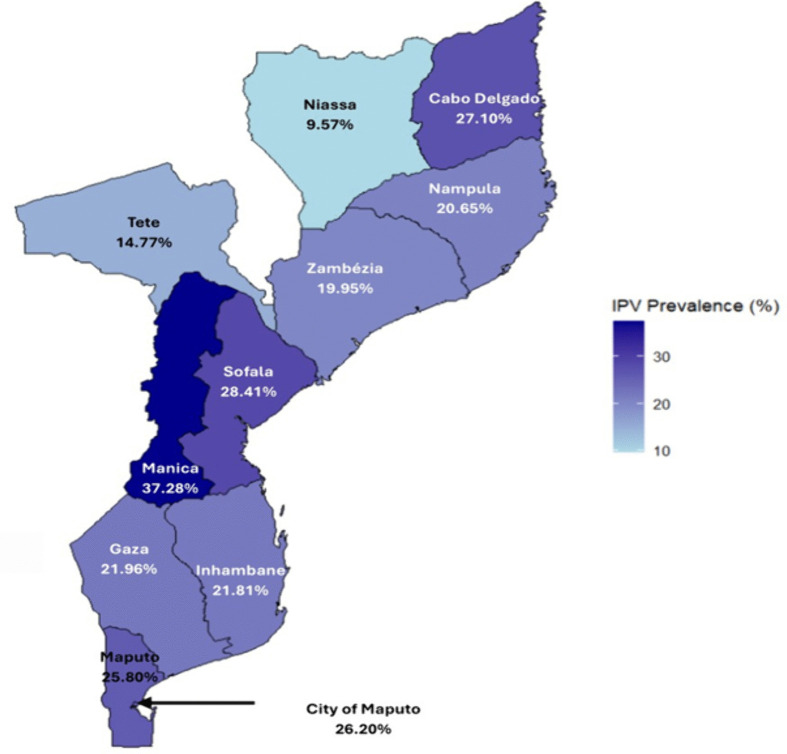
Provincial variation in the prevalence of lifetime intimate partner violence (pIPV) among women in Mozambique, as reported in the 2022-2023 DHS study (N = 4813). Results highlight the highest prevalence in Manica and the lowest in Niassa. Map generated using open-source geographic data from rnaturalearth (Natural Earth) and IPV prevalence data from DHS 2023. This figure complies with PLOS ONE’s CC BY 4.0 license.

Nearly 1 in 4 (21.92%) women reported that they had experienced physical abuse from a current or former partner since the age of 15 (**Fig 1**). This prevalence is similar to the pIPV experienced within the preceding 12 months (20.32%) (**S3 Fig**). Fig 2 shows the breakdown of regional variation in Lifetime pIPV across all provinces. Manica had the highest rate at 37.28%, followed by Cabo Delgado at 27.10% and Sofala at 28.41%. The lowest prevalence was found in Tete at 14.77% and Niassa at 9.57%.

The different types of pIPV experienced by women in Mozambique were assessed (**S4 Table**). The most commonly reported form was slapping (16.21%), followed by pushing, shaking, or having something thrown (8.37%). More severe acts of pIPV, such as being strangled or burnt (1.91%) or attacked with a weapon (1.20%), were less frequently reported. These findings highlight variations in the severity of pIPV, with milder forms being more prevalent compared to severe physical assault.

### Individual-level factors associated with the prevalence of lifetime pIPV

Several individual-level factors were significantly associated with Lifetime pIPV in this study (**Table 2**). Marital status was a strong determinant; women with a partner having a 61.7% higher likelihood of experiencing Lifetime pIPV compared to those who had never been in a union (aOR 1.617, 95% CI 1.293, 2.022). Women who are no longer with a partner had 78.7% higher odds of Lifetime pIPV (aOR: 1.787, 95% CI 1.377, 2.320). Education also played a role, women with higher than secondary education reporting decreased odds of Lifetime pIPV (aOR: 0.656, 95% CI 0.455, 0.945). Employment status was another key factor; employed women had a 33.9% increased likelihood of experiencing Lifetime pIPV compared to those not employed (aOR: 1.339, 95% CI 1.138, 1.576). Justification for beating was positively associated with Lifetime pIPV; women expressing moderate degree of justification having 40.8% higher odds of Lifetime pIPV (aOR: 1.408, 95% CI 1.116, 1.776) compared to those who did not justify violence. Husband/partner’s alcohol consumption was one of the strongest predictors. Husbands/partner’s alcohol drinking nearly tripled the odds of Lifetime pIPV (aOR: 2.928, 95% CI 2.491, 3.441).

**Fig 3:**
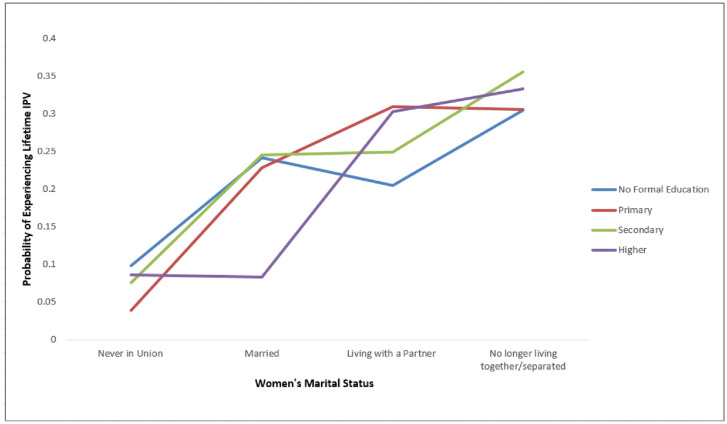
Interaction plot showing the predicted probabilities of experiencing Lifetime pIPV across education levels and marital statuses. Higher education reduces the likelihood of Lifetime pIPV, especially for married women, while those living with a partner or separated show consistently higher probabilities of pIPV.

An effect modification involving women’s educational level and marital status was also observed. Women with primary education who were living with a partner (aOR: 1.554, 95% CI 1.180, 2.048 had significantly higher odds of Lifetime pIPV compared to those with no formal education who had never been in a union. On the other hand, women with higher education and married (aOR: 0.195, 95% CI 0.0650, 0.584) had lower odds of Lifetime pIPV. Fig 3 further illustrates the interaction between women’s educational level and marital status and the associated probabilities of Lifetime pIPV.

For women with no formal education, those who have never been in a union show the lowest probability of pIPV, while the highest probabilities are seen in those living with a partner or no longer living together. Among women with primary education, pIPV probabilities are similar for those who are married or living with a partner, ranging from 0.22 to 0.30, while the gap between these groups and those never in union becomes more distinct. In secondary education, the probability of pIPV increases slightly for married or partnered women, with separated women showing the highest pIPV likelihood. For women with higher education, the probability of pIPV decreases significantly for married individuals, but remains relatively high, around 0.30–0.33, for those living with a partner or separated. Overall, being married appears to reduce the probability of pIPV as education increases, while the risks for those living with a partner or separated remain elevated across all education levels.

### Context-level factors associated with the prevalence of lifetime pIPV

The province of residence significantly impacted Lifetime pIPV prevalence. Women from Cabo Delgado (aOR: 1.640, 95% CI 1.162, 2.315) and Manica (aOR: 2.660, 95% CI 1.897, 3.732) were at substantially higher odds of experiencing Lifetime pIPV than those in Maputo. Conversely, women from Niassa (aOR: 0.445, 95% CI 0.293, 0.670), Inhambane (aOR: 0.679, [95% CI: 0.473, 0.974]), and Gaza (aOR: 0.570, 95% CI 0.393, 0.828) were less likely to experience pIPV. No other context-level variables were significantly associated with Lifetime pIPV. The Hosmer-Lemeshow test indicated a good fit for the logistic regression model (χ² = 4.75, p = 0.784).

## Discussion

Our study explored the prevalence and determinants of Lifetime pIPV among women in Mozambique using data from the 2022–2023 Mozambique DHS. The study highlights key individual-level factors influencing pIPV prevalence, including marital status, employment, justification of violence, and husband/partner’s alcohol consumption. We also found a significant effect modifier involving marital status and education level. On a contextual level, the study revealed regional disparities in pIPV prevalence with women in Cabo Delgado and Manica reporting the highest pIPV experiences.

Our study also revealed that cohabitating and separated women face significantly higher odds of experiencing lifetime pIPV compared to those never in a union. However, married women do not show a statistically significant increased risk, suggesting that the protective or risk-enhancing effects of marriage may be context-dependent. These findings align with the literature on this topic, suggesting that cohabiting women may face greater pIPV risks due to lower legal protections and economic instability, while separated women may experience retaliatory violence from former partners [3,7,25]. Studies generally show that IPV rates are higher among women with husbands/partners because men often use physical violence as a means to discipline as well as assert power and dominance over their wives/female partners [25,26]. Furthermore, women’s excessive reliance on their husbands/partners, in traditional Sub-Saharan African settings, further contributes to the high prevalence of IPV in certain cultures [7,26].

Our findings indicate that education alone was not significantly associated with IPV risk in the multivariable model. While higher education is often considered a protective factor due to increased autonomy, access to information, and financial independence [4,7], our results suggest that education’s effect may depend on the broader relationship context rather than acting as a direct protective factor. In many settings across Africa, educated women may still face pIPV if societal and gender norms do not shift alongside their educational advancements [8,25,27]. Although education did not show a direct association with pIPV in our model, the interaction between education and marital status, discussed later, suggests that pIPV risk is shaped by how education intersects with relationship dynamics.

Unlike education, employment was significantly associated with increased odds of experiencing IPV. Employed women had higher odds of IPV compared to unemployed women. This aligns with previous studies showing that while employment can empower women financially, it may also lead to power struggles in relationships where male dominance is expected [3,4]. In patriarchal contexts, men may perceive women’s financial independence as a threat to their traditional provider role, leading to increased tensions and IPV risk [9,12]. However, in societies where gender norms are more egalitarian, employment can serve as a protective factor by enhancing women’s economic and social bargaining power [11].

Consistent with existing literature, this study found that women’s justification of physical abuse significantly raised their odds of experiencing pIPV [7,15]. One study estimates that women’s justification of physical abuse increases their likelihood of experiencing pIPV by 30% while another study estimates a 57% higher odds of experiencing physical abuse [7,25]. A woman’s acceptance and justification of physical abuse are shaped by community attitudes that favor physical violence while dismissing female victimization. This context and environment not only increases the likelihood of being a victim but also pressures IPV victims to remain silent and accept the abuse [4,15].

Another strong predictor of Lifetime IPV is a husband/partner’s alcohol consumption. We found that women whose husbands/partners consumed alcohol were more than twice as likely to experience pIPV in their lifetime than those whose husbands/partners did not drink. Other studies have shown even stronger evidence that a husband/partner’s alcohol abuse increases the risk of experiencing abuse [27–29]. For instance, Olagbuji and colleagues found that having a partner who consumes alcohol raises the odds of experiencing IPV by 11 times [29]. Some sources suggest that alcohol consumption or addiction may lead men to neglect their families, increasing tensions in their intimate relationships that could result in physical abuse [7]. Others argue that alcohol consumption triggers immediate biological changes in men that lead to increased aggression and abuse toward their partners [18].

Notably, the effect modification between marital status and education highlights how the intersection of these two individual-level factors can either heighten or mitigate the likelihood of experiencing pIPV. This finding emphasizes the importance of recognizing how multiple dimensions of a woman’s identity and social position, intersect to shape her vulnerability to pIPV in Mozambique. Higher educational attainment in women is typically linked to greater autonomy and resource access, which can reduce IPV risk [30,31]. However, in the context of strong traditional gender norms, educated women in intimate partner relationships might still have greater vulnerability to experiencing IPV as their greater autonomy and independence challenge societal roles [32]. An intersectional approach provides a deeper understanding of these complexities and helps target effective interventions and policies.

On the contextual level, pIPV prevalence varied by province, with Cabo Delgado and Manica having the highest statistics, and Inhambane and Gaza having the lowest. This disparity in pIPV prevalence could be related to unequal wealth distribution across the country. According to a 2018 World Bank report, southern provinces like Inhambane, Gaza, Maputo, and Maputo City have smaller wealth distribution gaps between rural and urban areas and have a more even distribution of basic services, compared to others like Cabo Delgado, Manica, and Niassa [33]. Thus, unequal wealth and resource distribution in some provinces can worsen economic hardship, power imbalances, and psychological strain in relationships, increasing women’s vulnerability to IPV in their lifetime.

While many studies on IPV in sub-Saharan Africa have identified common predictors such as marital status, education, employment, justification for beating, and alcohol consumption, our findings extend beyond these well-established risk factors in several ways. First, we highlight provincial disparities in pIPV risk, with Cabo Delgado and Manica showing notably higher prevalence, emphasizing the need for region-specific interventions. Second, we identify an interaction between education and marital status, illustrating that education’s effect on IPV risk is not uniform but is shaped by relationship context. This nuance is often overlooked in broader SSA analyses. Third, by utilizing the most recent 2022–2023 Mozambique DHS data, this study provides an updated understanding of pIPV trends compared to many SSA studies relying on older datasets. Finally, our findings are framed within Mozambique’s unique sociocultural and policy context, particularly regarding gender norms, economic power dynamics, and alcohol consumption, reinforcing the importance of localized interventions.

### Strengths and limitations

The biggest strength of this study is that it utilized the most current data from a large, nationally representative dataset to provide a detailed analysis of Lifetime pIPV prevalence, making our results generalizable to all women between 15 and 49 years in Mozambique. Additionally, our analysis effectively examined both individual- and contextual-level factors, therefore offering a holistic view of the phenomenon of pIPV in the country. Furthermore, the study identified significant effect modification results between marital status and education, contributing to the understanding of how multiple dimensions of a woman’s identity affect pIPV prevalence. Again, our study highlights important provincial variations in pIPV prevalence, helping to identify high-risk areas of pIPV that will guide tailored interventions accordingly.

Despite these strengths, some limitations exist. For example, the cross-sectional nature of the study prevents us from establishing a causal relationship between the independent variables and Lifetime pIPV. Due to the stigma regarding pIPV, some women may refuse to disclose their experience of abuse, leading to underreporting, and potentially skewing the final results. Furthermore, the use of self-report questionnaires could lead to non-differential misclassification and/or recall bias. Finally, our results pertain specifically to pIPV and may not fully capture the burden of sexual or emotional violence against women in Mozambique therefore limiting our full understanding on IPV within the Mozambican context.

## Conclusion

Our study utilized the Mozambique DHS 2022–2023 data to examine the current prevalence of Lifetime pIPV in the country and to identify the specific individual and contextual factors contributing to it. Our findings showed that almost 1 in 4 women experienced pIPV in their lifetime. Marital status emerged as a key factor, with women who are cohabitating, or separated being at the highest odds of experiencing pIPV in their lives. Current employment also correlated with increased pIPV prevalence. Furthermore, the justification of violence significantly influenced pIPV prevalence. Similarly, husbands/partners’ consumption of alcohol was strongly associated with Lifetime pIPV prevalence. On a contextual level, provincial disparities in Lifetime pIPV were evident, with notably higher pIPV estimates in Cabo Delgado and Manica. Finally, the interaction between education and marital status underscores that the effect of education on pIPV risk varies by marital status.

Given the higher pIPV risk among cohabitating and separated women, targeted legal protections and support mechanisms should be strengthened to ensure their safety. Community-based interventions should focus on shifting harmful gender norms through educational campaigns and engaging men in pIPV prevention efforts. Additionally, integrating pIPV screening and response services within maternal and reproductive health programs can improve early detection and support for survivors. Finally, economic empowerment programs, such as microfinance initiatives or vocational training, should be designed to provide financial security while incorporating pIPV awareness and support systems.

## Supporting information

S1 Fig The theoretical framework of physical violence against women during pregnancy. Four different levels contribute to the risk of physical violence during pregnancy. Level 1 includes contextual factors, Level 2 includes both a woman and her husband/partners socio-demographic indicators, Level 3 encompasses the dynamics within the relationship between a woman and her husband/partner, and Level 4 contains factors associated with the woman’s attitudes and intentions towards physical abuse. Adapted from intimate partner violence and unintended pregnancy framework by Azevêdo et al., (2013).
(PDF)

S2 Table Details about independent variables and their recode status from Mozambique 20223−2023 demographic and health survey dataset. (PDF)

S3 Fig The prevalence of intimate partner violence (IPV) in the last 12 months among women in Mozambique, based on the 2022–2023 demographic and health survey, with a prevalence of 21.34% (N = 4813). (PDF)

S4 Table Types of physical intimate partner violence experienced by women in Mozambique. Demographic and Health Survey, 2022–2023, Mozambique.
(PDF)

S5 Table Results from bivariate analyses between all independent variables and history of intimate partner violence. Demographic and Health Survey, 2022–2023, Mozambique.
(PDF)

S6 File SAS analysis code used for data preparation and statistical analyses. (PDF)
