# Supplementary material for: Lifetime physical intimate partner violence (pIPV) among Mozambican women: Individual and contextual level factors driving its prevalence
Source: PLoS One. 2025 Dec 15;20(12):e0312640. doi: 10.1371/journal.pone.0312640 (PMC12704884; doi:10.1371/journal.pone.0312640)
Supplement: S1 Fig — Four different levels contribute to the risk of physical violence during pregnancy. Level 1 includes contextual factors, Level 2 includes both a woman and her husband/partners socio-demographic indicators, Level 3 encompasses the dynamics within the relationship between a woman and her husband/partner, and Level 4 contains factors associated with the woman’s attitudes and intentions towards physical abuse. Adapted from intimate partner violence and unintended pregnancy framework by Azevêdo et al., (2013). (PDF) [file pone.0312640.s001.pdf]

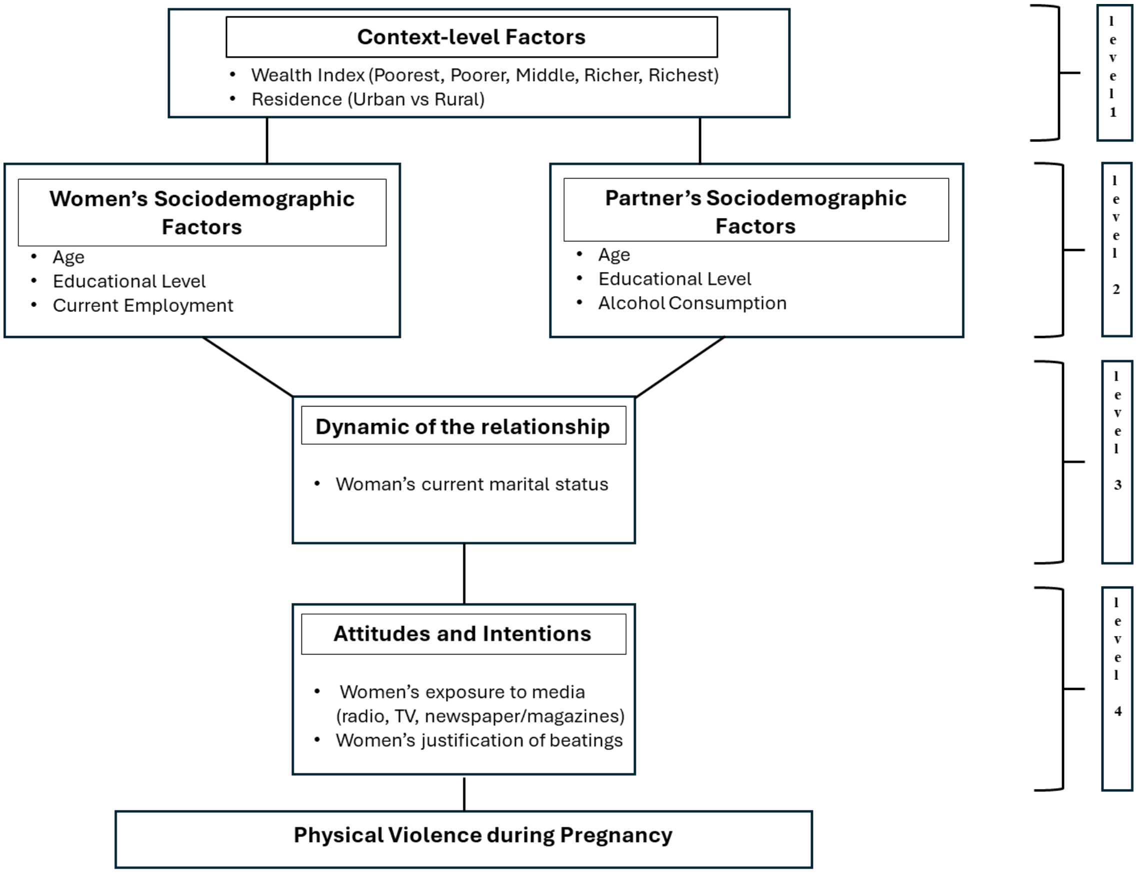

S1 Figure. The theoretical framework of physical violence against women during pregnancy. Four different levels contribute to the risk of physical violence during pregnancy. Level 1 includes contextual factors, Level 2 includes both a woman and her husband/partners socio-demographic indicators, Level 3 encompasses the dynamics within the relationship between a woman and her husband/partner, and Level 4 contains factors associated with the woman's attitudes and intentions towards physical abuse. Adapted from intimate partner violence and unintended pregnancy framework by Azevêdo et al., (2013).
