# Supplementary material for: Lifetime physical intimate partner violence (pIPV) among Mozambican women: Individual and contextual level factors driving its prevalence
Source: PLoS One. 2025 Dec 15;20(12):e0312640. doi: 10.1371/journal.pone.0312640 (PMC12704884; doi:10.1371/journal.pone.0312640)
Supplement: S2 Table — (PDF) [file pone.0312640.s002.pdf]

S2 Table. Details about Independent Variables and their Recode Status from Mozambique 20223-2023 Demographic and Health Survey Dataset

Proposed Independent Variable	Corresponding DHS Question	Variable Type	To be Re-coded?
Individual-Level Variables			
Maternal Age	/V013 "Age in 5-year groups"	Categorical: 15-19; 20-24; 25-29; 30-34; 35-39; 40-44; 45-49	Yes: Re-coded as '15–24', '25–34', '35–44' and '45 and above'
Current Marital Status	/V501 "Current marital status"	Categorical: Never in union; Married; Living with partner; Widowed; Divorced; No longer living together/separated	Yes: Re-code as 'married', 'Never in Union', 'Living with a Partner' 'No longer living together/separated
Husband/Partner's Age	/V730 "Husband/partner's age"	Continuous	Yes: Re-coded as '15–24', '25–34', '35–44' and '45 and above'
Husband/Partner's Level of Education	/V701 "Husband/partner's education level"	Categorical: No education; Primary; Secondary; Higher; Don't know	No
Maternal Educational Level	/V106 "Highest educational level"	Categorical: No education; Primary; Secondary; Higher	No
Maternal Current Employment Status	/V714 "Respondent currently working"	Categorical: No; Yes	No
Maternal Frequency of Access to Media (Radio, Print, Television)	/V157 "Frequency of reading newspaper or magazine"<br>/V158 "Frequency of listening to radio"<br>/V159 "Frequency of watching television"	Categorical: Not at all; Less than once a week; At least once a week; Almost every day	Yes: Re-coded 'Less than Once a Week', 'At Least Once a Week'
Husband/Partner's alcohol consumption	/D113 "Husband/partner drinks alcohol"	Categorical: No; Yes	No
Maternal Justification for physical Violence	/V744A "Beating justified if wife goes out without telling husband"<br>/V744B "Beating justified if wife neglects the children"<br>/V744C "Beating justified if wife argues with husband"	Categorical: No; Yes; I don't know	Yes: Re-coded as 'No justification' 'Moderate Justification' 'Moderate-to-complete Justification'

	/V744D "Beating justified if wife refuses to have sex with husband"<br>/V744E "Beating justified if wife burns the food"		
Age Gap between Respondent and Husband/Partner	/V730 - V012/<br>/V730 "Husband/partner's age"<br>/V012 "Respondent's current age"	Continuous	Yes: Re-coded as 'Husband's Younger' 'No Age Difference' 'Husband 1 – 4 Years Older' 'Husband 5 – 9 Years Older' 'Husband 10+ Years Older'
Woman's Religion	/V130 "Religion"	Categorical: Catholic; Islamic; Zion; Evangelical/Pentecostal; Anglican; No religion	Yes: Re-coded as 'No Specified Religion' 'Catholic' 'Other Christian' 'Muslim'
Sex of Household Head	/V151 "Sex of household head"	Categorical: Male; Female	No
Household Size	/V002 "Household number"	Continuous	Yes: Re-coded as 'Single Member' '2 – 3 Members' '4 – 5 Members' '6 – 7 Members' '8+ Members'
Polygamy	/V505 "Number of other wives"	Categorical: 0; 1; 2; 3; 4; 5; 7; 9	Yes: Re-coded as 'No Other Wife' '1 Other Wife' '2+ Other Wives'
Husband/Partner's Current Employment Status	/V704 "Husband/partner's occupation"	Categorical: Not working and didn't work in last 12 months; Jobs in the Army; Specialist jobs; Public Service Jobs; Technical Jobs; Vendor Jobs; Other Jobs; Retired/Students	Yes: Re-coded as 'No' 'Yes'
Context-Level Variables			
Wealth Index	/V190 "Wealth index combined"	Quintiles: Poorest; Poorer; Middle; Richer; Richest	No
Place of Residence	/V102 "Type of place of residence"	Categorical: Rural; Urban	No
Province	/V024 "Region"	Categorical: Niassa; Cabo Delgado;	No

		Nampula; Zambezia; Tete; Manica; Sofala; Inhambane; Gaza; Maputo; Cidade de Maputo	
