# Supplementary material for: Lifetime physical intimate partner violence (pIPV) among Mozambican women: Individual and contextual level factors driving its prevalence
Source: PLoS One. 2025 Dec 15;20(12):e0312640. doi: 10.1371/journal.pone.0312640 (PMC12704884; doi:10.1371/journal.pone.0312640)
Supplement: S3 Fig — (PDF) [file pone.0312640.s003.pdf]

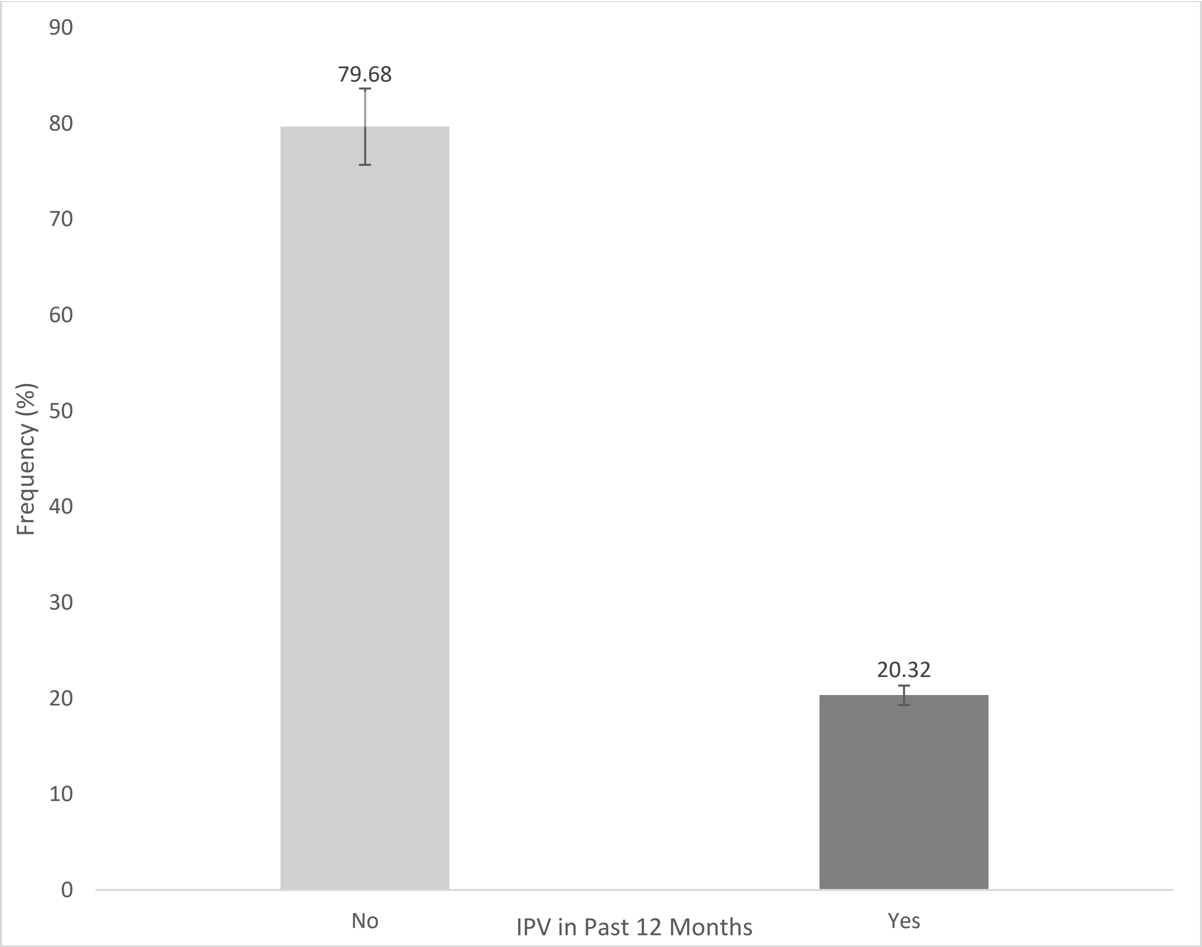

S3 Figure. The prevalence of Intimate Partner Violence (IPV) in the last 12 months among women in Mozambique, based on the 2022-2023 Demographic and Health Survey, with a prevalence of 21.34% (N=4813)
