# Supplementary material for: Lifetime physical intimate partner violence (pIPV) among Mozambican women: Individual and contextual level factors driving its prevalence
Source: PLoS One. 2025 Dec 15;20(12):e0312640. doi: 10.1371/journal.pone.0312640 (PMC12704884; doi:10.1371/journal.pone.0312640)
Supplement: S4 Table — Demographic and Health Survey, 2022–2023, Mozambique. (PDF) [file pone.0312640.s004.pdf]

S4 Types of Physical Intimate Partner Violence Experienced by Women in Mozambique. Demographic and Health Survey, 2022-2023, Mozambique

Variable (N=4,454)	Frequency (N)	Percentage (%)
Ever been pushed, shook or had something thrown by husband/partner	361.91	8.37
Ever been slapped by husband/partner	700.98	16.21
Ever been punched with fist or hit by something harmful by husband/partner	273.09	6.31
Ever been kicked or dragged by husband/partner	261.27	6.04
Ever been strangled or burnt by husband/partner	82.65	1.91
Ever been attacked with knife/gun or other weapon by husband/partner	51.69	1.20
Ever had arm twisted or hair pulled by husband/partner	165.07	3.82
Previous husband: ever hit, slap, kick or physically hurt respondent	235.87	5.46

¶Weighted frequencies derived using DHS survey weights
