# Supplementary material for: Lifetime physical intimate partner violence (pIPV) among Mozambican women: Individual and contextual level factors driving its prevalence
Source: PLoS One. 2025 Dec 15;20(12):e0312640. doi: 10.1371/journal.pone.0312640 (PMC12704884; doi:10.1371/journal.pone.0312640)
Supplement: S5 Table — Demographic and Health Survey, 2022–2023, Mozambique. (PDF) [file pone.0312640.s005.pdf]

S5 Table. Results from Bivariate Analyses Between All Independent Variables and History of Intimate Partner Violence. Demographic and Health Survey, 2022-2023, Mozambique

	**History of IPV**	
**Independent Variables**	**%Yes [95% CI]**	**p-value**
**Woman's Age**		**<0.0001****
15 – 24	6.23 [5.31,7.153]	
25 – 34	7.82[6.90,8.73]	
35 – 44	5.79[4.91,6.68]	
≥ 45	2.08[1.54,2.63]	
**Husband/Partner's Age**		0.1679
15 – 24	1.63[1.20,2.06]	
25 – 34	5.29[4.50,6.07]	
≥ 35	15.00[13.67,16.34]	
**Woman's Marital Status**		**<0.0001****
Never in Union	1.35[0.77,1.92]	
Married	6.26 [5.32,7.20	
Living with a Partner	9.64[8.68,10.60]	
No longer living together/separated	4.67 [3.87,5.47]	
**Woman's Educational Level**		**0.0031***
No Formal Education	6.23[5.25,7.21]	
Primary	10.31[9.21,11.41]	
Secondary	4.98 [4.27, 5.69]	
Higher	0.41[0.23,0.594]	
**Husband/Partner's Educational Level**		0.2078
No Formal Education	5.37[4.51,6.24]	
Primary	12.48 [11.24,13.72]	
Secondary Education or Higher	4.07 [3.30,4.83]	
**Woman's Current Employment Status**		**<0.0001****
No	13.33[11.99,14.66]	
Yes	8.60[7.70,9.49]	
**Woman's Access to Media**		0.3277
Less than Once a Week	14.08[12.73,15.43]	
At Least Once a Week	7.84[6.98,8.71]	

**Woman's Justification for Beating**		**0.0005***
No justification	17.29[15.92,18.66]	
Moderate Justification	2.40[1.91,2.88]	
Moderate-to-complete Justification	2.24[1.57,2.90]	
**Husband/Partner's Alcohol Consumption**		**<0.0001****
No	12.35[11.06,13.65]	
Yes	9.57[8.61,10.53]	
**Age Gap between Respondent and Husband/Partner**		**<0.0001****
Husband's Younger	6.68 [5.69,7.67]	
No Age Difference	0.49 [0.24,0.74]	
Husband 1 – 4 Years Older	5.54 [4.75,6.32]	
Husband 5 – 9 Years Older	5.31 [4.52,6.10]	
Husband 10+ Years Older	3.91 [3.21,4.61]	
**Woman's Religion**		**0.0002***
No Specified Religion	1.98 [1.52,2.44]	
Catholic	6.27 [5.22,7.33]	
Other Christian	10.31 [9.34,11.29]	
Muslim	3.36 [2.67,4.05]	
**Sex of Household Head**		0.5382
Male	15.02 [13.71,16.32]	
Female	6.91 [5.96,7.85]	
**House Hold Size**		0.4760
Single Member	0.28 [0.11,0.45]	
2 – 3 Members	0.46 [0.27,0.65]	
4 – 5 Members	0.49 [0.29,0.69]	
6 – 7 Members	1.71 [1.29,2.13]	
8+ Members	20.02 [18.53,21.51]	
**Polygamy**		**0.0280***
No Other Wives	19.40 [17.69,21.11]	
1 Other Wife	3.61 [2.84,4.38]	
2+ Wives	0.97 [0.42,1.51]	
**Husband/Partner's Current Employment Status**		**0.0013***
No	5.83 [4.68,6.97]	
Yes	18.34 [16.69,19.98]	
**Wealth Index**		0.7869

Poorest	4.52 [3.67,5.37]	
Poorer	3.76 [3.02,4.50]	
Middle	4.15[3.47,4.83]	
Richer	4.63[3.83,5.43]	
Richest	4.86[4.21,5.52]	
**Type of Place of Residence**		0.6531
Rural	13.65[12.354,14.96]	
Urban	8.27[7.33,9.21]	
**Province of Residence**		**<0.0001****
Niassa	0.68[0.45,0.92]	
Cabo Delgado	1.37[1.09,1.65]	
Nampula	4.86[3.89,5.83]	
Zambezia	3.20[2.34,4.06]	
Tete	2.29[1.80,2.79]	
Manica	2.46[2.01,2.92]	
Sofala	1.90[1.52,2.29]	
Inhambane	0.87[0.65,1.09]	
Gaza	0.69[0.50,0.89]	
Maputo	2.42[1.91,2.93]	
Cidade de Maputo	1.16[0.91,1.40]	

Statistically significant Chi-square results <0.0001

* Statistically significant Chi-square results <0.005*

[a]Wealth Index: Composite score derived from participants' household assets using principal component analysis

¶Weighted frequencies derived using DHS survey weights
