# Supplementary material for: Lifetime physical intimate partner violence (pIPV) among Mozambican women: Individual and contextual level factors driving its prevalence
Source: PLoS One. 2025 Dec 15;20(12):e0312640. doi: 10.1371/journal.pone.0312640 (PMC12704884; doi:10.1371/journal.pone.0312640)
Supplement: S6 File — (PDF) [file pone.0312640.s006.pdf]

```
libname IPV3 "C:\Users\mmuos\OneDrive\Graduate School\UMASS\All
Semesters\Summer 2024\Nazeem Project\DHS Study\Work in Progress\IPV3";

data IPV_p;
set IPV3.revised_ipv2;
run;

/***Coding outcome variable: Ever Experienced IPV**/
data IPV_p;
    set IPV_p;
    HistIPV = 0;
    if D105A in (1, 2, 3, 4) then HistIPV = 1;
    if D105B in (1, 2, 3, 4) then HistIPV = 1;
    if D105C in (1, 2, 3, 4) then HistIPV = 1;
    if D105D in (1, 2, 3, 4) then HistIPV = 1;
    if D105E in (1, 2, 3, 4) then HistIPV = 1;
    if D105F in (1, 2, 3, 4) then HistIPV = 1;
    if D105G in (1, 2, 3, 4) then HistIPV = 1;
    if D105J in (1, 2, 3, 4) then HistIPV = 1;
    if D130A in (1, 2, 3, 4) then HistIPV = 1;
run;

/**Prevalence of IPV in cohort selected***/
data IPV_p;
    set IPV_p;
    /* Adjust weight variable to be used in analysis */
    weight_adjusted = D005 / 1000000;
run;
proc freq data=IPV_p;
 weight weight_adjusted;
table HistIPV;
run;

/** Prevalence of IPV in the past 12 months in cohort selected **/
data IPV_p;
    set IPV_p;
    IPV_12mo = 0; /* Initialize IPV_12mo to 0 */

    /* Check conditions and set IPV_12mo to 1 if any condition is met */
    if D105A in (1, 2, 3, 4) or
       D105B in (1, 2, 3, 4) or
       D105C in (1, 2, 3, 4) or
       D105D in (1, 2, 3, 4) or
       D105E in (1, 2, 3, 4) or
       D105F in (1, 2, 3, 4) or
       D105G in (1, 2, 3, 4) or
       D105J in (1, 2) or
       D117A in (1, 2) or
       D130A = 1 then
       IPV_12mo = 1;
run;
proc freq data=IPV_p;
table IPV_12mo;
run;
proc freq data=IPV_p;
weight weight_adjusted;
table IPV_12mo;
```

```
run;

/***Determining the individual frequencies of physical abuse***/

data IPV_p;
    set IPV_p;
    /* Adjust weight variable to be used in analysis */
    weight_adjusted = D005 / 1000000;
      run;
proc freq data=IPV_p;
weight weight_adjusted;
Table D105A D105B D105C D105D D105E
D105F D105G D105J D117A D130A;
run;

/***Determining socio-demographic frequencies**/
/***1. Re-coding new variable: "Age"***/
data IPV_p;
    set IPV_p;
    Age = .; /* Initialize Age with a missing value */
    if V013=1 or V013=2 then Age=1;
    else if V013 = 3 or V013=4 then Age=2;
    else if V013 = 5 or V013 = 6 then Age = 3;
    else if V013 = 7 then Age = 4;
run;

/***1b. Frequency of Age***/
/**Prevalence of IPV in cohort selected***/
data IPV_p;
    set IPV_p;
    /* Adjust weight variable to be used in analysis */
    weight_adjusted = D005 / 1000000;
run;
proc freq data=IPV_p;
 weight weight_adjusted;
Table Age;
run;

/***2. Re-coding new variable: "Marital_Stat***/
data IPV_p;
    set IPV_p;
   if V501=0 then Marit_stat = 1;
   else if V501=1 then Marit_stat = 2;
    else if V501=2 then Marit_stat = 3;
    else if V501=3 or V501=4 or V501=5 then Marit_stat = 4;
run;

data IPV_p;
set IPV_p;
if Marit_stat = 1 then Marit_st = "No Union";
else if Marit_stat= 2 then Marit_st = "Married";
else if Marit_stat = 3 then Marit_st = "Living with a Partner";
else if Marit_stat = 4 then Marit_st = "Separated";
run;

proc logistic data=IPV_p;
    class Marit_st (ref="No Union");
```

```
    model Hist_IPV(event='1') = Marit_st;
run;

/***2b. Frequency of Marital Status***/
/**Prevalence of IPV in cohort selected***/
data IPV_p;
    set IPV_p;
    /* Adjust weight variable to be used in analysis */
    weight_adjusted = D005 / 1000000;
      run;
proc freq data=IPV_p;
weight weight_adjusted;
Table Marit_stat;
run;

/***3. Re-code into new variable: "Hub_age"***/
data IPV_p;
    set IPV_p;
    if 15 <= V730 <= 24 then Hubage = 1;
    else if 25 <= V730 <= 34 then Hubage = 2;
    else if 35 <= V730 <= 44 then Hubage = 3;
    else if V730 >= 45 then Hubage = 4;
run;

/**3b. Frequency of new variable: "Hub_age"***/
data IPV_p;
    set IPV_p;
    /* Adjust weight variable to be used in analysis */
    weight_adjusted = D005 / 1000000;
      run;
proc freq data=IPV_p;
weight weight_adjusted;
table Hubage;
run;

/*** 4. Recode new variable: Hub_edu ***/
data IPV_p;
    set IPV_p;
    if V701 = 0 then Hubedu = 0;
    else if V701 = 1 then Hubedu = 1;
    else if V701 = 2 then Hubedu = 2;
    else if V701 = 3 then Hubedu = 3;
    else if V701 = 8 then Hubedu = 0;
run;

/***4b. Frequency of Husband/Partner educational level***/
data IPV_p;
    set IPV_p;
    /* Adjust weight variable to be used in analysis */
    weight_adjusted = D005 / 1000000;
      run;
proc freq data=IPV_p;
weight weight_adjusted;
table Hubedu;
run;

/*** 5. Frequency for Maternal educational level***/
```

```
data IPV_p;
    set IPV_p;
    /* Adjust weight variable to be used in analysis */
    weight_adjusted = D005 / 1000000;
      run;
proc freq data=IPV_p;
weight weight_adjusted;
table V106;
run;

/***6. Frequency for Maternal current employment***/
data IPV_p;
    set IPV_p;
    /* Adjust weight variable to be used in analysis */
    weight_adjusted = D005 / 1000000;
      run;
proc freq data=IPV_p;
weight weight_adjusted;
table V714;
run;

/***7. Re-code into new variable: "Med_access"**/
data IPV_p;
    set IPV_p;
    Med_Access = .; /* Initialize Med_Access with a missing value */

    /* Check if any variable has a value of 2 or 3 */
    if V157 in (2, 3) or V158 in (2, 3) or V159 in (2, 3) then Med_Access =
1;
    /* Check if any variable has a value of 0 or 1 */
    else if V157 in (0, 1) or V158 in (0, 1) or V159 in (0, 1) then
Med_Access = 0;
run;

/***7b. Frequency of new variable: "Med_access***/
data IPV_p;
    set IPV_p;
    /* Adjust weight variable to be used in analysis */
    weight_adjusted = D005 / 1000000;
      run;
proc freq data=IPV_p;
weight weight_adjusted;
table Med_Access;
run;

/**8. Re-code V744A to V7444E to combine "No" and "I do not know"***/
data IPV_p;
    set IPV_p;
    if V744A = 0 or V744A = 8 then V744A_rev = 0;
    else if V744A = 1 then V744A_rev = 1;
    else V744A_rev = .; /* Optional: handle other cases, if necessary */
run;


data IPV_p;
    set IPV_p;
    if V744B = 0 or V744A = 8 then V74B_rev = 0;
```

```
    else if V744B = 1 then V744B_rev = 1;
    else V744B_rev = .; /* Optional: handle other cases, if necessary */
run;

/**8. Re-code V744A to V7444E to combine "No" and "I do not know"***/
data IPV_p;
    set IPV_p;
    if V744C = 0 or V744A = 8 then V744C_rev = 0;
    else if V744C = 1 then V744C_rev = 1;
    else V744C_rev = .; /* Optional: handle other cases, if necessary */
run;

/**8. Re-code V744A to V7444E to combine "No" and "I do not know"***/
data IPV_p;
    set IPV_p;
    if V744D = 0 or V744A = 8 then V744D_rev = 0;
    else if V744D = 1 then V744D_rev = 1;
    else V744D_rev = .; /* Optional: handle other cases, if necessary */
run;

data IPV_p;
    set IPV_p;
    if V744E = 0 or V744E = 8 then V744E_rev = 0;
    else if V744E = 1 then V744E_rev = 1;
    else V744E_rev = .; /* Optional: handle other cases, if necessary */
run;

/***8b. Re-code new variable: "Phys_just" - Remember that this is a scale
from 0 to 5***/
data IPV_p;
set IPV_p;
Beat_Just = sum(of V744A_rev, V744B_rev, V744C_rev, V744D_rev, V744E_rev);
run;

/***8c. Frequency of new variable: "Beat_Just"***/
data IPV_p;
    set IPV_p;
    /* Adjust weight variable to be used in analysis */
    weight_adjusted = D005 / 1000000;
      run;

proc freq data=IPV_p;
weight weight_adjusted;
table Beat_Just;
run;

/**9.Re-coding Beat_Just into fewer categories (0 = No justifications;
1=moderate; 2=moderate to complete justification)***/
data IPV_p;
    set IPV_p;
    Beat_justb = .; /* Initialize Beat_justb with a missing value */
    if Beat_Just = 0 then Beat_justb = 0;
    else if Beat_Just in (1, 2) then Beat_justb = 1;
    else if Beat_Just in (3, 4, 5) then Beat_justb = 2;
run;

/*9b. Frequency for updated Justifications for beatings***/
```

```
data IPV_p;
    set IPV_p;
    /* Adjust weight variable to be used in analysis */
    weight_adjusted = D005 / 1000000;
      run;
proc freq data=IPV_p;
weight weight_adjusted;
table Beat_justb;
run;

/***10. Frequency of variable: Wealth Index***/
data IPV_p;
    set IPV_p;
    /* Adjust weight variable to be used in analysis */
    weight_adjusted = D005 / 1000000;
      run;
proc freq data=IPV_p;
weight weight_adjusted;
table V190;
run;

/***10a. Frequency of variable: Wealth Index (Rural/Urban)***/
proc freq data=IPV_p;
weight weight_adjusted;
table V190A;
run;

/***11. Frequency of variable: Place of residence***/
data IPV_p;
    set IPV_p;
    /* Adjust weight variable to be used in analysis */
    weight_adjusted = D005 / 1000000;
      run;
proc freq data=IPV_p;
weight weight_adjusted;
table V102;
run;

/***12. Frequency of variable: Current Province***/
data IPV_p;
    set IPV_p;
    /* Adjust weight variable to be used in analysis */
    weight_adjusted = D005 / 1000000;
      run;
proc freq data=IPV_p;
weight weight_adjusted;
table V024;
run;

/***13. Frequency of variable: Husband Drinking***/
data IPV_p;
    set IPV_p;
    /* Adjust weight variable to be used in analysis */
    weight_adjusted = D005 / 1000000;
      run;
proc freq data=IPV_p;
weight weight_adjusted;
```

```
table D113;
run;

/***14. Frequency of variable: Age difference***/

/**14a. Compute Age Gap***/
data IPV_p;
set IPV_p;
Age_Gap = v730 - v012;
run;

  /**14b.  Categorize Age Gap ***/
     /* Define Age_Gap_Cat as a Character Variable */
    LENGTH Age_Gap_Cat $25;

    /* Categorize Age Gap */
      data IPV_p;
      set IPV_p;
    IF Age_Gap < 0 THEN Age_Gap_Cat = "Husband Younger";
    ELSE IF Age_Gap = 0 THEN Age_Gap_Cat = "Same Age";
    ELSE IF 1 <= Age_Gap <= 4 THEN Age_Gap_Cat = "Husband 1-4 yrs Older";
    ELSE IF 5 <= Age_Gap <= 9 THEN Age_Gap_Cat = "Husband 5-9 yrs Older";
    ELSE IF Age_Gap >= 10 THEN Age_Gap_Cat = "Husband 10+ yrs Older";
    ELSE Age_Gap_Cat = "Missing";

RUN;

/**14.c Obtain frequencies of Age_gap**/
/* Check Frequencies */
data IPV_p;
    set IPV_p;
    /* Adjust weight variable to be used in analysis */
    weight_adjusted = D005 / 1000000;
      run;

PROC FREQ DATA=IPV_p;
weight  weight_adjusted;
    TABLE Age_Gap_Cat / MISSING;
RUN;

/***15. Frequency of Religion***/

/**15a. Re-categorize religion variable***/
data IPV_p;
    set IPV_p;
    Religion = .; /* Initialize Beat_justb with a missing value */
    if V130 in (6, 96) then Religion = 0;
    else if V130 in (3, 4, 5) then Religion = 1;
    else if V130 in (2) then Religion = 2;
      else if V130 in (1) then Religion = 3;
run;


/*/* 15b. Check Frequencies */
data IPV_p;
    set IPV_p;
    /* Adjust weight variable to be used in analysis */
```

```
    weight_adjusted = D005 / 1000000;
      run;
PROC FREQ DATA=IPV_p;
weight weight_adjusted;
    TABLE Religion / MISSING;
RUN;*/


/**16. Frequency of Sex of Household Head**/
PROC FREQ DATA=IPV_p;
data IPV_p;
    set IPV_p;
    /* Adjust weight variable to be used in analysis */
    weight_adjusted = D005 / 1000000;
      run;
      proc freq data=IPV_p;
      weight weight_adjusted;
    TABLE V151 / MISSING;
RUN;

/***17. Distribution of Household size***/

/**17a. Re-categorize the household variable from continuous to
categorical***/
DATA IPV_p;
    SET IPV_p;  /* Replace with your actual dataset name */

    /* Create Household Size Category Variable */
    LENGTH HousHold_Size $15;  /* Define the length of the new variable */

    /* Assign categories based on household size */
    IF V002 = 1 THEN HousHold_Size = "Single Member";
    ELSE IF 2 <= V002 <= 3 THEN HousHold_Size = "2-3 Members";
    ELSE IF 4 <= V002 <= 5 THEN HousHold_Size = "4-5 Members";
    ELSE IF 6 <= V002 <= 7 THEN HousHold_Size = "6-7 Members";
    ELSE IF V002 >= 8 THEN HousHold_Size = "8+ Members";
    ELSE HousHold_Size = "Missing";

RUN;

/***17b. Frequency of HousHold_Size**/
data IPV_p;
    set IPV_p;
    /* Adjust weight variable to be used in analysis */
    weight_adjusted = D005 / 1000000;
      run;
PROC FREQ DATA=IPV_p;
weight  weight_adjusted;
    TABLE HousHold_Size / MISSING;
RUN;

/***18. Frequency of polygamy***/

proc freq data=IPV_p;
table V505;
run;
```

```
/**Recategorize variable**/
data IPV_p;
    set IPV_p;
    Polygamy = .; /* Initialize Beat_justb with a missing value */
    if V505 in (0) then Polygamy = 0;
    else if V505 in (1) then Polygamy = 1;
    else if V505 in (2, 3, 4, 7, 9) then Polygamy = 2;
run;
data IPV_p;
    set IPV_p;
    /* Adjust weight variable to be used in analysis */
    weight_adjusted = D005 / 1000000;
      run;
PROC FREQ DATA=IPV_p;
weight weight_adjusted;
    TABLE Polygamy / MISSING;
RUN;


/**Frequency of Husband's Occupation**/
data IPV_p;
    set IPV_p;
    Husb_job = .;
    if V704 in (0, 99997) then Husb_job = 0;
    if V704 in (1, 2, 3, 11, 12, 13, 14, 21, 22, 23,
24, 25, 26, 31, 32, 33, 34, 35, 41, 42, 43, 44,
51, 52, 53, 54, 61, 62, 63, 71, 72, 73, 74, 75
81, 82, 83, 91, 92, 93, 94,
95, 96, 99996) then Husb_job = 1;
run;

proc freq data=IPV_p;
weight weight_adjusted;
table Husb_job;
run;

/**Rerun Outcome variable**/

data IPV_p;
    set IPV_p;
    Hist_IPV = 0;
    if D105A in (1, 2, 3, 4) then Hist_IPV = 1;
    if D105B in (1, 2, 3, 4) then Hist_IPV = 1;
    if D105C in (1, 2, 3, 4) then Hist_IPV = 1;
    if D105D in (1, 2, 3, 4) then Hist_IPV = 1;
    if D105E in (1, 2, 3, 4) then Hist_IPV = 1;
    if D105F in (1, 2, 3, 4) then Hist_IPV = 1;
    if D105G in (1, 2, 3, 4) then Hist_IPV = 1;
    if D105J in (1, 2, 3, 4) then Hist_IPV = 1;
    if D130A in (1, 2, 3, 4) then Hist_IPV = 1;
run;

/***DATA ANALYSES SECTION.
A)Bivariate analyses between each independent variable and outcome variable

1. Age and Hist_IPV***/
```

```
proc surveyfreq data=IPV_p;
weight weight_adjusted;
    tables Age*Hist_IPV / chisq cl;
    title 'Crosstabulation of Age and Hist_IPV with Chi-Square Test and 95%
Confidence Intervals';
run;

/**2. Marit_Stat and IPV***/
proc surveyfreq data=IPV_p;
weight weight_adjusted;
    tables Marit_stat*HistIPV / chisq cl;
    title 'Crosstabulation of Marit_stat and Hist_IPV with Chi-Square Test
and 95% Confidence Intervals';
run;

/**3. Hub_age and IPV**/
/***3. Re-code into new variable: "Hub_age" since last category has
insufficient cells***/
data IPV_p;
    set IPV_p;
    if 15 <= V730 <= 24 then Hubage = 1;
    else if 25 <= V730 <= 34 then Hubage = 2;
    else if V730 >= 35 then Hubage = 3;
run;
/**Looking for patterns of missingness for Hubedu***/
proc freq data=IPV_p;
weight weight_adjusted;
    tables Hubedu*HistIPV / missing;
run;

/**Compare with complete cases***/
proc freq data=IPV_p;
    tables HistIPV / missing;
run;

proc freq data=IPV_p;
    where not missing(Hubedu);
    tables HistIPV;
run;
/***The output from the above suggests that the pattern of missingness
affects the distribution of IPV prevalence across Hubage levels. Therefore,
imputation needed***/
proc freq data=IPV_p;
weight weight_adjusted;
    tables Hubage / missing;
    title 'Frequency Distribution of Hubage'; /**To determine the mode**/
run;
proc freq data=IPV_p;
table V704;
RUN;


data IPV_p;
    set IPV_p;
    /* Create a flag for missing Hubage */
    if missing(Hubage) then Hubage_missing_flag = 1;
    else Hubage_missing_flag = 0;
```

```
    /* Impute missing Hubage with the mode value (e.g., 3) */
    if missing(Hubage) then Hubage = 3; /* Replace 3 with the actual mode
value */
run;

proc surveyfreq data=IPV_p;
weight weight_adjusted;
    tables Hubage*HistIPV / chisq cl;
    title 'Crosstabulation of Husband Age and Hist_IPV with Chi-Square Test
and 95% Confidence Intervals';
run;

/**4. Husband Educational level and IPV since last category has insufficient
cells**/

/**Recode new variable: Hubedu ***/
data IPV_p;
    set IPV_p;
    if V701 = 0 then Hubedu = 0;
    else if V701 = 1 then Hubedu = 1;
    else if V701 = 2 then Hubedu = 2;
    else if V701 = 3 then Hubedu = 2;
    else if V701 = 8 then Hubedu = 0;
run;
/***The output  suggests that the pattern of missingness affects the
distribution of IPV prevalence across Hubedu levels. Therefore,
imputation needed***/
proc freq data=IPV_p;
    tables Hubedu / missing;
    title 'Frequency Distribution of Hubedu'; /**To determine the mode**/
run;
data IPV_p;
    set IPV_p;
    /* Create a flag for missing Hubage */
    if missing(Hubedu) then Hubedu_missing_flag = 1;
    else Hubedu_missing_flag = 0;

    /* Impute missing Hubage with the mode value (e.g., 1) */
    if missing(Hubedu) then Hubedu = 1; /* Replace 1 with the actual mode
value */
run;

proc surveyfreq data=IPV_p;
weight weight_adjusted;
    tables Hubedu*HistIPV / chisq cl;
    title 'Crosstabulation of Husband educational level and Hist_IPV with
Chi-Square Test and 95% Confidence Intervals';
run;

/***5. Maternal educational level and IPV***/
proc surveyfreq data=IPV_p;
weight weight_adjusted;
    tables V106*Hist_IPV / chisq cl;
    title 'Crosstabulation of Maternal educational level and Hist_IPV with
Chi-Square Test and 95% Confidence Intervals';
run;
```

```
/***6. Maternal current employment and IPV**/

proc surveyfreq data=IPV_p;
weight weight_adjusted;
    tables V714*Hist_IPV / chisq cl;
    title 'Crosstabulation of Maternal current employment and Hist_IPV with
Chi-Square Test and 95% Confidence Intervals';
run;

/***7. Media access and IPV***/
proc surveyfreq data=IPV_p;
weight weight_adjusted;
    tables Med_access*Hist_IPV / chisq cl;
    title 'Crosstabulation of Media access and Hist_IPV with Chi-Square Test
and 95% Confidence Intervals';
run;

/**8. Justification for physical abuse and IPV**/
proc surveyfreq data=IPV_p;
weight weight_adjusted;
    tables Beat_justb*Hist_IPV / chisq cl;
    title 'Crosstabulation of Justification for physical abuse and Hist_IPV
with Chi-Square Test and 95% Confidence Intervals';
run;

/**9. Wealth Index and IPV***/
proc surveyfreq data=IPV_p;
weight weight_adjusted;
    tables V190*Hist_IPV / chisq cl;
    title 'Crosstabulation of Wealth Index and Hist_IPV with Chi-Square Test
and 95% Confidence Intervals';
run;

/**10. Place of Residence and IPV**/
proc surveyfreq data=IPV_p;
weight weight_adjusted;
    tables V102*Hist_IPV / chisq cl;
    title 'Crosstabulation of Place of residence and Hist_IPV with Chi-Square
Test and 95% Confidence Intervals';
run;

/***11. Province of residence and IPV**/

proc surveyfreq data=IPV_p;
weight weight_adjusted;
    tables V024*Hist_IPV / chisq cl;
    title 'Crosstabulation of Province of residence and Hist_IPV with Chi-
Square Test and 95% Confidence Intervals';
run;

/***12. Husband drinking and IPV**/
/***The output  suggests that the pattern of missingness affects the
distribution of IPV prevalence across Husband drinking levels. Therefore,
imputation needed***/
proc freq data=IPV_p;
weight weight_adjusted;
```

```
    tables D113 / missing;
    title 'Frequency Distribution of Husband Drinking'; /**To determine the
mode**/
run;
data IPV_p;
    set IPV_p;
    /* Create a flag for missing Husband drinking */
    if missing(D113) then D113_missing_flag = 1;
    else D113_missing_flag = 0;

    /* Impute missing Hubage with the mode value (e.g., 1) */
    if missing(D113) then D113 = 0; /* Replace 1 with the actual mode value
*/
run;

proc surveyfreq data=IPV_p;
weight weight_adjusted;
    tables D113*HistIPV / chisq cl;
    title 'Crosstabulation of Husband drinking and Hist_IPV with Chi-Square
Test and 95% Confidence Intervals';
run;

/**13. Age gap and IPV***/
proc surveyfreq data=IPV_p;
weight weight_adjusted;
    tables Age_Gap_Cat*Hist_IPV / chisq cl;
    title 'Crosstabulation of Husband drinking and Hist_IPV with Chi-Square
Test and 95% Confidence Intervals';
run;

/**14. Religion and IPV**/
proc surveyfreq data=IPV_p;
weight weight_adjusted;
    tables Religion*Hist_IPV / chisq cl;
    title 'Crosstabulation of Husband drinking and Hist_IPV with Chi-Square
Test and 95% Confidence Intervals';
run;

/**15. Sex of Household Head and IPV**/

proc surveyfreq data=IPV_p;
weight weight_adjusted;
    tables V151*Hist_IPV / chisq cl;
    title 'Crosstabulation of Husband drinking and Hist_IPV with Chi-Square
Test and 95% Confidence Intervals';
run;

/***16. Household Size and IPV**/

proc surveyfreq data=IPV_p;
weight weight_adjusted;
    tables HousHold_Size*Hist_IPV / chisq cl;
    title 'Crosstabulation of Husband drinking and Hist_IPV with Chi-Square
Test and 95% Confidence Intervals';
run;

/***17. Polygamy and IPV***/
```

```
proc surveyfreq data=IPV_p;
weight weight_adjusted;
    tables Polygamy*Hist_IPV / chisq cl;
    title 'Crosstabulation of Husband drinking and Hist_IPV with Chi-Square
Test and 95% Confidence Intervals';
run;

/***18. Husband Job and IPV**/
proc surveyfreq data=IPV_p;
weight weight_adjusted;
    tables Husb_job*Hist_IPV / chisq cl;
    title 'Crosstabulation of Husband drinking and Hist_IPV with Chi-Square
Test and 95% Confidence Intervals';
run;

/**Multivariate analyses between
A)individual-level variables and out come variable - Using Stepwise approach
(Media Access Included despite > threshold (0.20)***/

proc logistic data=IPV_p;
    class Age (ref='1') Hubage (ref='1') Marit_stat (ref='0') Hubedu
(ref='0') V106 (ref='0') V714 (ref='0') Med_Access (ref='0') Beat_justb
(ref='0') D113 (ref='0');
    model HistIPV(event='1') = Age Hubage Marit_stat Hubedu V106 V714
Med_Access Beat_justb D113 / clodds=pl lackfit;
    oddsratio Age;
    oddsratio Hubage;
      oddsratio Marit_stat;
    oddsratio Hubedu;
    oddsratio V106;
    oddsratio V714;
      oddsratio Med_Access;
    oddsratio Beat_justb;
    oddsratio D113;
      run;

      proc logistic data=IPV_p;
    class Age (ref='1') Hubage (ref='1') Marit_stat (ref=FIRST) Hubedu
(ref='0') V106 (ref='0') V714 (ref='0') Beat_justb (ref='0') D113 (ref='0');
    model HistIPV(event='1') = Age Hubage Marit_stat Hubedu V106 V714
Beat_justb D113 / clodds=pl lackfit;
    oddsratio Age;
    oddsratio Hubage;
      oddsratio Marit_stat;
    oddsratio Hubedu;
    oddsratio V106;
    oddsratio V714;
    oddsratio Beat_justb;
    oddsratio D113;
      run;

/*A-1)individual-level variables and outcome variable - Using Stepwise
approach (Media Access Excluded)***/

proc logistic data=IPV_p;
```

```
    class Age (ref='1') Hubage (ref='1') Marit_st (ref="No Union") Hubedu
(ref='0') V106 (ref='0') V714 (ref='0') Beat_justb (ref='0') D113 (ref='0');
    model HistIPV(event='1') = Age Hubage Marit_st Hubedu V106 V714
Beat_justb D113 / clodds=pl lackfit;
    oddsratio Age;
    oddsratio Hubage;
      oddsratio Marit_st;
    oddsratio Hubedu;
    oddsratio V106;
    oddsratio V714;
    oddsratio Beat_justb;
    oddsratio D113;
      run;

      proc freq data=IPV_p;
    tables Marit_st Age Hubage Hubedu V106 V714 Beat_justb D113 / missing;
run;
/**Imputing missing data - Husband data**/
/* Calculate mode for Hubedu */ proc freq data=IPV_p noprint; tables Hubedu /
out=modeHubedu (keep=Hubedu count); run;
/* Calculate mode for Hubage */ proc freq data=IPV_p noprint; tables Hubage /
out=modeHubage (keep=Hubage count); run;
/* Print mode values */ proc print data=modeHubedu; run;
proc print data=modeHubage; run; data _null_; set modeHubedu; call
symputx('mode_Hubedu', Hubedu); stop; run;
data _null_; set modeHubage; call symputx('mode_Hubage', Hubage); stop; run;
%put &=mode_Hubedu; %put &=mode_Hubage; data IPV_p; set IPV_p;
/* Impute missing values using mode values */ if missing(Hubedu) then Hubedu
= &mode_Hubedu; if missing(Hubage) then Hubage = &mode_Hubage; run; proc
means data=IPV_p nmiss; var Hubedu Hubage; run;
/* Impute missing values with a placeholder (e.g., '99') */
data IPV_p; set IPV_p;
/* Impute missing values with a placeholder value */ if missing(Hubedu) then
Hubedu = '99'; if missing(Hubage) then Hubage = '99'; run;

/*A-2)individual-level variables and outcome variable - Using Stepwise
approach (Media Access and Husband Education Excluded)***/
proc logistic data=IPV_p;
    class Age (ref='1') Hubedu (ref='1') Hubage (ref='1') Marit_st (ref="No
Union") V106 (ref='0') V714 (ref='0') Beat_justb (ref='0') D113 (ref='0');
    model HistIPV(event='1') = Age Hubage Hubedu Marit_st V106 V714
Beat_justb D113 / clodds=pl lackfit;
    oddsratio Age;
    oddsratio Hubage;
      oddsratio Marit_st;
      oddsratio Hubedu;
    oddsratio V106;
    oddsratio V714;
    oddsratio Beat_justb;
    oddsratio D113;
      run;

/***From the above, A-2 seems to be the best model (AIC and HL Goodness of
Fit)***/

/**B)Contextual-level variables and outcome variable - Using Stepwise
approach (With Wealth Index Although Wealth Index > Threshold (0.20)***/
```

```
proc logistic data=IPV_p;
    class V190 (ref='1') V024 (ref='10') V102 (ref='1');
    model HistIPV(event='1') = V190 V024 V102 / clodds=pl lackfit;
    oddsratio V190;
    oddsratio V024;
    oddsratio V102;
   run;

/**B-1)Contextual-level variables and outcome variable - Using Stepwise
approach (Wealth Index Excluded)***/
proc logistic data=IPV_p;
    class V024 (ref='10') V102 (ref='1');
    model HistIPV(event='1') = V024 V102 / clodds=pl lackfit;
    oddsratio V024;
    oddsratio V102;
   run;
   /**The above output shows that the models are comparable and not that
different hence Wealth Index will be included moving forward***/

  proc format;
    value MaritFmt
        0 = "Never Married"
        1 = "Married"
        2 = "Cohabiting"
        3 = "Separated/Divorced";
run;
data IPV_p;
    set IPV_p;
    format Marit_stat MaritFmt.; /* Apply format */
run;
proc freq data=IPV_p;
    tables Marit_stat / missing;
    format Marit_stat MaritFmt.; /* Ensure formatted values appear */
run;

proc freq data=IPV_p;
table Marit_stat;
run;
/**C)Both Individual and Contextual-level variables and outcome variable -
Using Stepwise approach***/

proc logistic data=IPV_p;
    class Age (ref='1')
          Hubage (ref='1')
          Marit_st (ref="No Union")  /* Match this exactly to PROC FREQ
output */
          V106 (ref='0')
          V714 (ref='0')
          Beat_justb (ref='0')
          D113 (ref='0')
          V024 (ref='10')
          V102 (ref='1');
model Hist_IPV(event='1') = Age Hubage Marit_st V106 V714 Beat_justb D113
V024 V102
        / clodds=pl lackfit;
    oddsratio Age;
```

```
    oddsratio Hubage;
    oddsratio Marit_st;
    oddsratio V106;
    oddsratio V714;
    oddsratio Beat_justb;
    oddsratio D113;
    oddsratio V024;
    oddsratio V102;
run;

/**C-1)Both Individual and Contextual-level variables and outcome variable -
Using Stepwise approach (Media Access excluded)***/
proc logistic data=IPV_p;
    class Age (ref='1') Hubage (ref='1') Marit_st (ref='No Union') V106
(ref='0')
          V714 (ref='0') Beat_justb (ref='0') D113 (ref='0') V024 (ref='10')
V102 (ref='1') Hubedu (ref='0');
    model HistIPV(event='1') = Age Hubage Hubedu Marit_stat V106 V714
Beat_justb D113 V024 V102 / clodds=pl lackfit;
    oddsratio Age;
    oddsratio Hubage;
    oddsratio Marit_stat;
    oddsratio V106;
    oddsratio V714;
    oddsratio Beat_justb;
    oddsratio D113;
    oddsratio V024;
    oddsratio V102;
    oddsratio Hubedu;
run;


/**C-2)Both Individual and Contextual-level variables and outcome variable -
Using Stepwise approach (Media Access and Husband Edu excluded)***/
proc logistic data=IPV_p;
    class Age (ref='1') Hubage (ref='1') Marit_stat (ref='0') V106 (ref='0')
V714 (ref='0') Beat_justb (ref='0') D113 (ref='0')
V190 (ref='1') V024 (ref='10') V102 (ref='1');
    model HistIPV(event='1') = Age Hubage Marit_stat V106 V714 Beat_justb
D113 V190 V024 V102/ clodds=pl lackfit;
    oddsratio Age;
    oddsratio Hubage;
      oddsratio Marit_stat;
    oddsratio V106;
    oddsratio V714;
    oddsratio Beat_justb;
    oddsratio D113;
    oddsratio V190;
    oddsratio V024;
    oddsratio V102;
      run;

/**C-3)Both Individual and Contextual-level variables and outcome variable -
Using Stepwise approach (Media Access and Wealth Index excluded)***/
proc logistic data=IPV_p;
    class Age (ref='1') Hubage (ref='1') Marit_st (ref='No Union') Hubedu
(ref='0') V106 (ref='0') V714 (ref='0') Beat_justb (ref='0') D113 (ref='0')
```

```
 V024 (ref='10') V102 (ref='1');
    model Hist_IPV(event='1') = Age Hubage Marit_st Hubedu V106 V714
Beat_justb D113  V024 V102/ clodds=pl lackfit;
    oddsratio Age;
    oddsratio Hubage;
      oddsratio Marit_st;
      oddsratio Hubedu;
    oddsratio V106;
    oddsratio V714;
    oddsratio Beat_justb;
    oddsratio D113;
    oddsratio V024;
    oddsratio V102;
      run;

/**C-4)Both Individual and Contextual-level variables and outcome variable -
Using Stepwise approach (Media Access, Hubedu, and Wealth Index excluded)***/
proc logistic data=IPV_p;
    class Age (ref='1') Hubage (ref='1') Marit_stat (ref='1') V106 (ref='0')
V714 (ref='0') Beat_justb (ref='0') D113 (ref='0')
 V024 (ref='10') V102 (ref='1');
    model HistIPV(event='1') = Age Hubage Marit_stat V106 V714 Beat_justb
D113  V024 V102/ clodds=pl lackfit;
    oddsratio Age;
    oddsratio Hubage;
      oddsratio Marit_stat;
    oddsratio V106;
    oddsratio V714;
    oddsratio Beat_justb;
    oddsratio D113;
    oddsratio V024;
    oddsratio V102;
      run;

      /**C-4a)Both Individual and Contextual-level variables and outcome
variable - Using Stepwise approach (Media Access, Hubedu, and Wealth Index
excluded)***/
proc logistic data=IPV_p;
    class Age (ref='1') Hubage (ref='1') Marit_stat (ref='1') V106 (ref='0')
V714 (ref='0') Beat_justb (ref='0') D113 (ref='0')
 V024 (ref='10') V102 (ref='1')Hubedu (ref='0');
    model HistIPV(event='1') = Age Hubage Hubedu Marit_stat V106 V714
Beat_justb D113  V024 V102/ clodds=pl lackfit;
    oddsratio Age;
    oddsratio Hubage;
      oddsratio Marit_stat;
    oddsratio V106;
    oddsratio V714;
    oddsratio Beat_justb;
    oddsratio D113;
    oddsratio V024;
    oddsratio V102;
      oddsratio Hubedu;
      run;

/**C-4b)Both Individual and Contextual-level variables and outcome variable -
Using Stepwise approach (Media Access and Wealth Index excluded)***/
```

```
proc logistic data=IPV_p;
    class Age (ref='1')
            Hubage (ref='1')
          Marit_stat (ref='1')
          V106 (ref='0')
          V714 (ref='0')
          Beat_justb (ref='0')
          D113 (ref='0')
          V024 (ref='10')
          V102 (ref='1');

    model HistIPV(event='1') = Age Hubage Marit_stat V106 V714 Beat_justb
D113 V024 V102
        / clodds=pl lackfit;

    oddsratio Age;
    oddsratio Hubage;
    oddsratio Marit_stat;
    oddsratio V106;
    oddsratio V714;
    oddsratio Beat_justb;
    oddsratio D113;
    oddsratio V024;
    oddsratio V102;
run;
/**C-4c)Both Individual and Contextual-level variables and outcome variable -
Using Stepwise approach (Media Access, Hubedu, and Wealth Index excluded)***/

proc logistic data=IPV_p;
    class Age (ref='1')
          Hubage (ref='1')
          Marit_stat (ref='1')
          V106 (ref='0')
          V714 (ref='0')
          Beat_justb (ref='0')
          D113 (ref='0')
          V024 (ref='10')
          V102 (ref='1')
          / param=glm; /* Ensures individual p-values for levels */

      weight weight_adjusted;

    model HistIPV(event='1') = Age Hubage Marit_stat V106 V714 Beat_justb
D113 V024 V102
        / clodds=pl lackfit;

    /* Get p-values for each variable's levels */
    oddsratio Age;
    oddsratio Hubage;
    oddsratio Marit_stat;
    oddsratio V106;
    oddsratio V714;
    oddsratio Beat_justb;
    oddsratio D113;
    oddsratio V024;
    oddsratio V102;
```

```
    /* Produces p-values for each level of each categorical variable */
    run;

    /* Get Type 3 Tests (Overall significance test for each variable) */
proc logistic data=IPV_p;
    class Age (ref='1')
          Hubage (ref='1')
          Marit_stat (ref='1')
          V106 (ref='0')
          V714 (ref='0')
          Beat_justb (ref='0')
          D113 (ref='0')
          V024 (ref='10')
          V102 (ref='1')
          / param=glm;

    weight weight_adjusted;

    model HistIPV(event='1') = Age Hubage Marit_stat V106 V714 Beat_justb
D113 V024 V102;

    /* Get p-values for each level */
    test Age;
    test Hubage;
    test Marit_stat;
    test V106;
    test V714;
    test Beat_justb;
    test D113;
    test V024;
    test V102;
run;


      /**C-5)Both Individual and Contextual-level variables and outcome
variable - Using Stepwise approach (Media Access, Hubedu,
      and Wealth Index excluded)NOTE: New Variables Added - Religion***/
proc logistic data=IPV_p;
    class Age (ref='1') Hubage (ref='1') Marit_st (ref='No Union') Hubedu
(ref='0') V106 (ref='0') V714 (ref='0') Beat_justb (ref='0') D113 (ref='0')
 V024 (ref='10') V102 (ref='1') Religion (ref='0');
    model Hist_IPV(event='1') = Age Hubage Marit_st Hubedu V106 V714
Beat_justb D113  V024 V102 Religion / clodds=pl lackfit;
    oddsratio Age;
    oddsratio Hubage;
      oddsratio Marit_st;
      oddsratio Hubedu;
    oddsratio V106;
    oddsratio V714;
      oddsratio Religion;
    oddsratio Beat_justb;
    oddsratio D113;
    oddsratio V024;
    oddsratio V102;
      run;
```

```
      /**C-6)Both Individual and Contextual-level variables and outcome
variable - Using Stepwise approach (Media Access, Hubedu,
      and Wealth Index excluded)NOTE: New Variables Added - Household
Size***/
proc logistic data=IPV_p;
    class Age (ref='1') Hubage (ref='1') Marit_st (ref='No Union') Hubedu
(ref='0') V106 (ref='0') V714 (ref='0') Beat_justb (ref='0') D113 (ref='0')
 V024 (ref='10') V102 (ref='1') Religion (ref='0')HousHold_Size (ref="Single
Member");
    model Hist_IPV(event='1') = Age Hubage Marit_st Hubedu V106 V714
Beat_justb D113  V024 V102 HousHold_Size / clodds=pl lackfit;
    oddsratio Age;
    oddsratio Hubage;
      oddsratio Marit_st;
      oddsratio Hubedu;
    oddsratio V106;
    oddsratio V714;
      oddsratio HousHold_Size;
    oddsratio Beat_justb;
    oddsratio D113;
    oddsratio V024;
    oddsratio V102;
      run;

      proc freq data=IPV_p;
      table Polygamy;
      run;

      /**C-7)Both Individual and Contextual-level variables and outcome
variable - Using Stepwise approach (Media Access, Hubedu,
      and Wealth Index excluded)NOTE: New Variables Added - Polygamy***/

      proc freq data=IPV_p noprint;
    tables Polygamy / out=modePolygamy (keep=Polygamy count);
run;

/* Print mode value */
proc print data=modePolygamy;
run;
data _null_;
    set modePolygamy;
    call symputx('mode_Polygamy', Polygamy);
    stop;
run;

/* Print stored mode value */
%put &=mode_Polygamy;
data IPV_p;
    set IPV_p;

    /* Replace missing values with the mode value */
    if missing(Polygamy) then Polygamy = &mode_Polygamy;

run;

/* Check if missing values are imputed */
proc means data=IPV_p nmiss;
```

```
    var Polygamy;
run;
data IPV_p;
    set IPV_p;

    /* Replace missing values with a placeholder */
    if missing(Polygamy) then Polygamy = 99;

run;

proc freq data=IPV_p;
weight weight_adjusted;
table Polygamy/missing;
run;


proc logistic data=IPV_p;
    class Age (ref='1') Hubage (ref='1') Marit_st (ref='No Union') Hubedu
(ref='0') V106 (ref='0') V714 (ref='0') Beat_justb (ref='0') D113 (ref='0')
 V024 (ref='10') V102 (ref='1') Polygamy (ref='0')HousHold_Size (ref="Single
Member");
    model Hist_IPV(event='1') = Age Hubage Marit_st Hubedu V106 V714
Beat_justb D113  V024 V102 Polygamy / clodds=pl lackfit;
    oddsratio Age;
    oddsratio Hubage;
      oddsratio Marit_st;
      oddsratio Hubedu;
    oddsratio V106;
    oddsratio V714;
      oddsratio Polygamy;
    oddsratio Beat_justb;
    oddsratio D113;
    oddsratio V024;
    oddsratio V102;
      run;

            /**C-8)Both Individual and Contextual-level variables and outcome
variable - Using Stepwise approach (Media Access, Hubedu,
      and Wealth Index excluded)NOTE: New Variables Added - Age Gap***/

      proc freq data=IPV_p;
      table Age_Gap_Cat;
      run;

      proc logistic data=IPV_p;
    class Age (ref='1') Hubage (ref='1') Marit_st (ref='No Union') Hubedu
(ref='0') V106 (ref='0') V714 (ref='0') Beat_justb (ref='0') D113 (ref='0')
 V024 (ref='10') V102 (ref='1') Age_Gap_Cat (ref="Husband
Younger")HousHold_Size (ref="Single Member");
    model Hist_IPV(event='1') = Age Hubage Marit_st Hubedu V106 V714
Beat_justb D113  V024 V102 Age_Gap_Cat / clodds=pl lackfit;
    oddsratio Age;
    oddsratio Hubage;
      oddsratio Marit_st;
      oddsratio Hubedu;
    oddsratio V106;
    oddsratio V714;
```

```
      oddsratio Age_Gap_Cat;
    oddsratio Beat_justb;
    oddsratio D113;
    oddsratio V024;
    oddsratio V102;
      run;

      /**C-9)Both Individual and Contextual-level variables and outcome
variable - Using Stepwise approach (Media Access, Hubedu,
      and Wealth Index excluded)NOTE: New Variables Added - Husband Job***/

/***Based on the above output, the model without media and wealth index is
the best for individual&Context-level factors model***/

      /**Imputation of Husb_job***/
proc freq data=IPV_p noprint;
    tables Husb_job / out=modeHusbJob (keep=Husb_job count);
run;

/* Print mode value */
proc print data=modeHusbJob;
run;
data _null_;
    set modeHusbJob;
    call symputx('mode_Husb_job', Husb_job);
    stop;
run;

/* Print stored mode value */
%put &=mode_Husb_job;
data IPV_p;
    set IPV_p;

    /* Replace missing values with the mode value */
    if missing(Husb_job) then Husb_job = &mode_Husb_job;

run;

/* Check if missing values are imputed */
proc means data=IPV_p nmiss;
    var Husb_job;
run;
data IPV_p;
    set IPV_p;

    /* Replace missing values with a placeholder */
    if missing(Husb_job) then Husb_job = 99;

run;

proc logistic data=IPV_p;
    class Age (ref='1') Hubage (ref='1') Marit_st (ref='No Union') Hubedu
(ref='0') V106 (ref='0') V714 (ref='0') Beat_justb (ref='0') D113 (ref='0')
 V024 (ref='10') V102 (ref='1') Husb_job (ref="0");
    model Hist_IPV(event='1') = Age Hubage Marit_st Hubedu V106 V714
Beat_justb D113  V024 V102 Husb_job / clodds=pl lackfit;
    oddsratio Age;
```

```
    oddsratio Hubage;
      oddsratio Marit_st;
      oddsratio Hubedu;
    oddsratio V106;
    oddsratio V714;
      oddsratio Husb_job;
    oddsratio Beat_justb;
    oddsratio D113;
    oddsratio V024;
    oddsratio V102;
      run;



/**D)Women's Sociodemographic variables and outcome variable - Using Stepwise
approach***/
      proc logistic data=IPV_p;
          class Age (ref='1') V106 (ref='0') V714 (ref='0');
          model HistIPV(event='1') = Age V106 V714/ clodds=pl lackfit;
          oddsratio Age;
          oddsratio V106;
          oddsratio V714;
            run;

/**E)Husband/Partner's Sociodemographic variables and outcome variable -
Using Stepwise approach***/

proc logistic data=IPV_p;
    class Hubage (ref='1')  D113 (ref='0');
    model HistIPV(event='1') = Hubage D113 / clodds=pl lackfit;
    oddsratio Hubage;
    oddsratio D113;
      run;

      /**The socio-demographic model is the weakest model from the group of
models p-value = 0.0312**/
      /**F)Attitude-level variables and outcome variable - Using Stepwise
approach***/

      proc logistic data=IPV_p;
    class Med_Access (ref='0') Beat_justb (ref='0');
    model HistIPV(event='1') = Med_Access Beat_justb / clodds=pl lackfit;
      oddsratio Med_Access;
    oddsratio Beat_justb;
      run;

/***INTERACTION CALCULATIONS BETWEEN VARIABLES IN COMPLETE MODEL (INDIVIDUAL
AND CONTEXT LEVEL)***/
      /** 1) AGE**

      A) Age X Partner's Age**/
proc logistic data=IPV_p;
    class Age (ref='1')
          Hubage (ref='1')
          Marit_stat (ref='0')
          Hubedu (ref='0')
          V106 (ref='0')
```

```
          V714 (ref='0')
          Beat_justb (ref='0')
          D113 (ref='0')
          V024 (ref='10')
          V102 (ref='1');
    model HistIPV(event='1') = Age|Hubage Marit_stat Hubedu V106 V714
Beat_justb D113 V024 V102 / clodds=pl lackfit;
    oddsratio Age;
    oddsratio Hubage;
    oddsratio Marit_stat;
    oddsratio Hubedu;
    oddsratio V106;
    oddsratio V714;
    oddsratio Beat_justb;
    oddsratio D113;
    oddsratio V024;
    oddsratio V102;
run;
/***This interaction term is not statistically significant (p-value =0.3455 >
0.05)

B) Age X Marital Status**/
proc logistic data=IPV_p;
    class Age (ref='1')
          Marit_stat (ref='0')
          Hubage (ref='1')
          Hubedu (ref='0')
          V106 (ref='0')
          V714 (ref='0')
          Beat_justb (ref='0')
          D113 (ref='0')
          V024 (ref='10')
          V102 (ref='1');
    model HistIPV(event='1') = Age|Marit_stat Hubage Hubedu V106 V714
Beat_justb D113 V024 V102 / clodds=pl lackfit;
    oddsratio Age;
    oddsratio Marit_stat;
       oddsratio Hubage;
    oddsratio Hubedu;
    oddsratio V106;
    oddsratio V714;
    oddsratio Beat_justb;
    oddsratio D113;
    oddsratio V024;
    oddsratio V102;
      run;

/***This interaction term is not statistically significant (p-value =0.4196 >
0.05)

C) Age X Maternal Education**/

proc logistic data=IPV_p;
    class Age (ref='1')
          Marit_stat (ref='0')
          Hubage (ref='1')
          Hubedu (ref='0')
```

```
          V106 (ref='0')
          V714 (ref='0')
          Beat_justb (ref='0')
          D113 (ref='0')
          V024 (ref='10')
          V102 (ref='1');
    model HistIPV(event='1') = Age|V106  Marit_stat Hubage Hubedu V714
Beat_justb D113 V024 V102 / clodds=pl lackfit;
    oddsratio Age;
      oddsratio V106;
    oddsratio Marit_stat;
       oddsratio Hubage;
    oddsratio Hubedu;
    oddsratio V714;
    oddsratio Beat_justb;
    oddsratio D113;
    oddsratio V024;
    oddsratio V102;
      run;
/***This interaction term is not statistically significant (p-value =0.9503 >
0.05)

D) Age X Partner's Education**/
proc logistic data=IPV_p;
    class Age (ref='1')
          Marit_stat (ref='0')
          Hubage (ref='1')
          Hubedu (ref='0')
          V106 (ref='0')
          V714 (ref='0')
          Beat_justb (ref='0')
          D113 (ref='0')
          V024 (ref='10')
          V102 (ref='1');
    model HistIPV(event='1') = Age|Hubedu V106 Marit_stat Hubage V714
Beat_justb D113 V024 V102 / clodds=pl lackfit;
    oddsratio Age;
      oddsratio Hubedu;
      oddsratio V106;
    oddsratio Marit_stat;
      oddsratio Hubage;
    oddsratio V714;
    oddsratio Beat_justb;
    oddsratio D113;
    oddsratio V024;
    oddsratio V102;
      run;

/***This interaction term is not statistically significant (p-value =0.9518 >
0.05)

E) Age X Current Employment**/

proc logistic data=IPV_p;
    class Age (ref='1')
          Marit_stat (ref='0')
          Hubage (ref='1')
```

```
          Hubedu (ref='0')
          V106 (ref='0')
          V714 (ref='0')
          Beat_justb (ref='0')
          D113 (ref='0')
          V024 (ref='10')
          V102 (ref='1');
    model HistIPV(event='1') = Age|V714 Hubedu V106 Marit_stat Hubage
Beat_justb D113 V024 V102 / clodds=pl lackfit;
    oddsratio Age;
      oddsratio V714;
      oddsratio Hubedu;
      oddsratio V106;
    oddsratio Marit_stat;
      oddsratio Hubage;
    oddsratio Beat_justb;
    oddsratio D113;
    oddsratio V024;
    oddsratio V102;
      run;

/***This interaction term is not statistically significant (p-value =0.4002 >
0.05)

F) Age X Justification of beatings**/
proc logistic data=IPV_p;
    class Age (ref='1')
          Marit_stat (ref='0')
          Hubage (ref='1')
          Hubedu (ref='0')
          V106 (ref='0')
          V714 (ref='0')
          Beat_justb (ref='0')
          D113 (ref='0')
          V024 (ref='10')
          V102 (ref='1');
    model HistIPV(event='1') = Age|Beat_justb V714 Hubedu V106 Marit_stat
Hubage D113 V024 V102 / clodds=pl lackfit;
    oddsratio Age;
      oddsratio Beat_justb;
      oddsratio V714;
      oddsratio Hubedu;
      oddsratio V106;
    oddsratio Marit_stat;
      oddsratio Hubage;
    oddsratio D113;
    oddsratio V024;
    oddsratio V102;
      run;
/***This interaction term is not statistically significant (p-value =0.3825 >
0.05)

G) Age X Partner's Alcoholism**/

proc logistic data=IPV_p;
    class Age (ref='1')
          Marit_stat (ref='0')
```

```
          Hubage (ref='1')
          Hubedu (ref='0')
          V106 (ref='0')
          V714 (ref='0')
          Beat_justb (ref='0')
          D113 (ref='0')
          V024 (ref='10')
          V102 (ref='1');
    model HistIPV(event='1') = Age|D113 Beat_justb V714 Hubedu V106
Marit_stat Hubage V024 V102 / clodds=pl lackfit;
    oddsratio Age;
      oddsratio D113;
      oddsratio Beat_justb;
      oddsratio V714;
      oddsratio Hubedu;
      oddsratio V106;
    oddsratio Marit_stat;
      oddsratio Hubage;
    oddsratio V024;
    oddsratio V102;
      run;
/***This interaction term is not statistically significant (p-value =0.3250 >
0.05)

H) Age X Province of Residence**/
proc logistic data=IPV_p;
    class Age (ref='1')
          Marit_stat (ref='0')
          Hubage (ref='1')
          Hubedu (ref='0')
          V106 (ref='0')
          V714 (ref='0')
          Beat_justb (ref='0')
          D113 (ref='0')
          V024 (ref='10')
          V102 (ref='1');
    model HistIPV(event='1') = Age|V024 D113 Beat_justb V714 Hubedu V106
Marit_stat Hubage V102 / clodds=pl lackfit;
    oddsratio Age;
      oddsratio V024;
      oddsratio D113;
      oddsratio Beat_justb;
      oddsratio V714;
      oddsratio Hubedu;
      oddsratio V106;
    oddsratio Marit_stat;
      oddsratio Hubage;
    oddsratio V102;
      run;

/***This interaction term is not statistically significant (p-value =0.6247 >
0.05)

I) Age X Place of Residence (Rural vs Urban) **/

proc logistic data=IPV_p;
    class Age (ref='1')
```

```
          Marit_stat (ref='0')
          Hubage (ref='1')
          Hubedu (ref='0')
          V106 (ref='0')
          V714 (ref='0')
          Beat_justb (ref='0')
          D113 (ref='0')
          V024 (ref='10')
          V102 (ref='1');
    model HistIPV(event='1') = Age|V102 V024 D113 Beat_justb V714 Hubedu V106
Marit_stat Hubage/ clodds=pl lackfit;
    oddsratio Age;
      oddsratio V102;
      oddsratio V024;
      oddsratio D113;
      oddsratio Beat_justb;
      oddsratio V714;
      oddsratio Hubedu;
      oddsratio V106;
    oddsratio Marit_stat;
      oddsratio Hubage;
      run;

/***This interaction term is not statistically significant (p-value =0.3971 >
0.05)

/** 2) Residence (Rural vs Urban)**
      A) Residence X Partner Age***/

proc logistic data=IPV_p;
    class Age (ref='1')
          Hubage (ref='1')
          Marit_stat (ref='0')
          Hubedu (ref='0')
          V106 (ref='0')
          V714 (ref='0')
          Beat_justb (ref='0')
          D113 (ref='0')
          V024 (ref='10')
          V102 (ref='1');
    model HistIPV(event='1') = Age Marit_stat Hubedu V106 V714 Beat_justb
D113 V024 V102|Hubage / clodds=pl lackfit;
    oddsratio Age;
    oddsratio Marit_stat;
    oddsratio Hubedu;
    oddsratio V106;
    oddsratio V714;
    oddsratio Beat_justb;
    oddsratio D113;
    oddsratio V024;
    oddsratio V102;
      oddsratio Hubage;
run;

/***This interaction term is not statistically significant (p-value =0.5904 >
0.05)
```

```
B) Residence X Maternal Education***/
proc logistic data=IPV_p;
    class Age (ref='1')
          Hubage (ref='1')
          Marit_stat (ref='0')
          Hubedu (ref='0')
          V106 (ref='0')
          V714 (ref='0')
          Beat_justb (ref='0')
          D113 (ref='0')
          V024 (ref='10')
          V102 (ref='1');
    model HistIPV(event='1') = Age Marit_stat Hubedu V714 Beat_justb D113
V024 V102|V106 Hubage / clodds=pl lackfit;
    oddsratio Age;
    oddsratio Marit_stat;
    oddsratio Hubedu;
    oddsratio V714;
    oddsratio Beat_justb;
    oddsratio D113;
    oddsratio V024;
    oddsratio V102;
      oddsratio V106;
      oddsratio Hubage;
run;
/***This interaction term is not statistically significant (p-value =0.8190 >
0.05)

C) Residence X Partner's Education***/
proc logistic data=IPV_p;
    class Age (ref='1')
          Hubage (ref='1')
          Marit_stat (ref='0')
          Hubedu (ref='0')
          V106 (ref='0')
          V714 (ref='0')
          Beat_justb (ref='0')
          D113 (ref='0')
          V024 (ref='10')
          V102 (ref='1');
    model HistIPV(event='1') = Age Marit_stat V714 Beat_justb D113 V024
V102|Hubedu V106 Hubage / clodds=pl lackfit;
    oddsratio Age;
    oddsratio Marit_stat;
    oddsratio V714;
    oddsratio Beat_justb;
    oddsratio D113;
    oddsratio V024;
    oddsratio V102;
      oddsratio Hubedu;
      oddsratio V106;
      oddsratio Hubage;
run;
/***This interaction term has BORDERLINE significance (p-value =0.0967 >
0.05)

D) Residence X Maternal Employment***/
```

```
proc logistic data=IPV_p;
    class Age (ref='1')
          Hubage (ref='1')
          Marit_stat (ref='0')
          Hubedu (ref='0')
          V106 (ref='0')
          V714 (ref='0')
          Beat_justb (ref='0')
          D113 (ref='0')
          V024 (ref='10')
          V102 (ref='1');
    model HistIPV(event='1') = Age Marit_stat Beat_justb D113 V024 V102|V714
Hubedu V106 Hubage / clodds=pl lackfit;
    oddsratio Age;
    oddsratio Marit_stat;
    oddsratio Beat_justb;
    oddsratio D113;
    oddsratio V024;
    oddsratio V102;
      oddsratio V714;
      oddsratio Hubedu;
      oddsratio V106;
      oddsratio Hubage;
run;

/***This interaction term is not statistically significant (p-value =0.9234 >
0.05)

E) Residence X Justification of Beatings***/

proc logistic data=IPV_p;
    class Age (ref='1')
          Hubage (ref='1')
          Marit_stat (ref='0')
          Hubedu (ref='0')
          V106 (ref='0')
          V714 (ref='0')
          Beat_justb (ref='0')
          D113 (ref='0')
          V024 (ref='10')
          V102 (ref='1');
    model HistIPV(event='1') = Age Marit_stat D113 V024 V102|Beat_justb V714
Hubedu V106 Hubage / clodds=pl lackfit;
    oddsratio Age;
    oddsratio Marit_stat;
    oddsratio D113;
    oddsratio V024;
    oddsratio V102;
      oddsratio Beat_justb;
      oddsratio V714;
      oddsratio Hubedu;
      oddsratio V106;
      oddsratio Hubage;
run;

/***This interaction term is not statistically significant (p-value =0.8801 >
0.05)
```

```
F) Residence X Partner's Alcoholism***/
proc logistic data=IPV_p;
    class Age (ref='1')
          Hubage (ref='1')
          Marit_stat (ref='0')
          Hubedu (ref='0')
          V106 (ref='0')
          V714 (ref='0')
          Beat_justb (ref='0')
          D113 (ref='0')
          V024 (ref='10')
          V102 (ref='1');
    model HistIPV(event='1') = Age Marit_stat V024 V102|D113 Beat_justb V714
Hubedu V106 Hubage / clodds=pl lackfit;
    oddsratio Age;
    oddsratio Marit_stat;
    oddsratio V024;
    oddsratio V102;
      oddsratio D113;
      oddsratio Beat_justb;
      oddsratio V714;
      oddsratio Hubedu;
      oddsratio V106;
      oddsratio Hubage;
run;

/***This interaction term is not statistically significant (p-value =0.7769 >
0.05)

G) Residence X Province of Residence***/
proc logistic data=IPV_p;
    class Age (ref='1')
          Hubage (ref='1')
          Marit_stat (ref='0')
          Hubedu (ref='0')
          V106 (ref='0')
          V714 (ref='0')
          Beat_justb (ref='0')
          D113 (ref='0')
          V024 (ref='10')
          V102 (ref='1');
    model HistIPV(event='1') = Age Marit_stat V102|V024 D113 Beat_justb V714
Hubedu V106 Hubage / clodds=pl lackfit;
    oddsratio Age;
    oddsratio Marit_stat;
    oddsratio V102;
      oddsratio V024;
      oddsratio D113;
      oddsratio Beat_justb;
      oddsratio V714;
      oddsratio Hubedu;
      oddsratio V106;
      oddsratio Hubage;
run;
```

```
/***This interaction term has BORDERLINE significance (p-value =0.0697 >
0.05)

H) Residence X Marital Status***/

proc logistic data=IPV_p;
    class Age (ref='1')
          Hubage (ref='1')
          Marit_stat (ref='0')
          Hubedu (ref='0')
          V106 (ref='0')
          V714 (ref='0')
          Beat_justb (ref='0')
          D113 (ref='0')
          V024 (ref='10')
          V102 (ref='1');
    model HistIPV(event='1') = Age V102|Marit_stat V024 D113 Beat_justb V714
Hubedu V106 Hubage / clodds=pl lackfit;
    oddsratio Age;
    oddsratio V102;
      oddsratio Marit_stat;
      oddsratio V024;
      oddsratio D113;
      oddsratio Beat_justb;
      oddsratio V714;
      oddsratio Hubedu;
      oddsratio V106;
      oddsratio Hubage;
run;

/***This interaction term is not statistically significant (p-value =0.7930 >
0.05)

/** 3) Women Education**

      A) Women Education X Partner Age***/

proc logistic data=IPV_p;
    class Age (ref='1')
          Hubage (ref='1')
          Marit_stat (ref='0')
          Hubedu (ref='0')
          V106 (ref='0')
          V714 (ref='0')
          Beat_justb (ref='0')
          D113 (ref='0')
          V024 (ref='10')
          V102 (ref='1');
    model HistIPV(event='1') = Age Marit_stat Hubedu V106|Hubage V714
Beat_justb D113 V024 V102 / clodds=pl lackfit;
    oddsratio Age;
    oddsratio Marit_stat;
    oddsratio Hubedu;
    oddsratio V106;
      oddsratio Hubage;
    oddsratio V714;
    oddsratio Beat_justb;
```

```
    oddsratio D113;
    oddsratio V024;
    oddsratio V102;
      run;

/***This interaction term is not statistically significant (p-value =0.8490 >
0.05)

      B) Women Education X Marital Status**/
proc logistic data=IPV_p;
    class Age (ref='1')
          Hubage (ref='1')
          Marit_st (ref='No Union')
          Hubedu (ref='0')
          V106 (ref='0')
          V714 (ref='0')
          Beat_justb (ref='0')
          D113 (ref='0')
          V024 (ref='10')
          V102 (ref='1');
             weight weight_adjusted;
    model HistIPV(event='1') = Age Hubedu V106*Marit_st Marit_st V106 Hubage
V714 Beat_justb D113 V024 V102 / clodds=pl lackfit;
    oddsratio Age;
    oddsratio Hubedu;
    oddsratio V106;
    oddsratio Marit_st;
    oddsratio Hubage;
    oddsratio V714;
    oddsratio Beat_justb;
    oddsratio D113;
    oddsratio V024;
    oddsratio V102;
      /* Produce Hosmer-Lemeshow Goodness-of-Fit Test */
    ods select LackFitPartition LackFitChiSq GlobalTests FitStatistics;
run;

/***This interaction term IS STATISTICALLY SIGNIFICANT (p-value =0.0078 <
0.05)**/

proc logistic data=IPV_p;
    class Age (ref='1') Hubage (ref='1') Marit_stat (ref='0') Hubedu
(ref='0') V106 (ref='0') V714 (ref='0') Beat_justb (ref='0') D113 (ref='0')
V024 (ref='10') V102 (ref='1');
    model HistIPV(event='1') = Age Hubedu V106 Marit_stat V106*Marit_stat
Hubage V714 Beat_justb D113 V024 V102 / clodds=pl;
    output out=pred_data pred=prob;  /* Changed PREDPROB=prob to PRED=prob */
run;

proc export data=pred_data
    outfile="C:\Users\mmuos\OneDrive\Graduate School\UMASS\All
Semesters\Summer 2024\Nazeem Project\DHS Study\Work in
Progress\IPV3\predicted_probabilities.xlsx"
    dbms=xlsx
    replace;
run;
```

```
      /**C) Women Education X Partner's Education**/

proc logistic data=IPV_p;
    class Age (ref='1')
          Hubage (ref='1')
          Marit_stat (ref='0')
          Hubedu (ref='0')
          V106 (ref='0')
          V714 (ref='0')
          Beat_justb (ref='0')
          D113 (ref='0')
          V024 (ref='10')
          V102 (ref='1');
    model HistIPV(event='1') = Age V106|Hubedu V106|Marit_stat Hubage V714
Beat_justb D113 V024 V102 / clodds=pl lackfit;
    oddsratio Age;
    oddsratio V106;
      oddsratio Hubedu;
      oddsratio Hubage;
      oddsratio Marit_stat;
      oddsratio V714;
    oddsratio Beat_justb;
    oddsratio D113;
    oddsratio V024;
    oddsratio V102;
      run;

/***This interaction term is not statistically significant (p-value =0.1325 >
0.05) when WomEducation|Marital_Stat is
      already in the model

      D) Women Education X Moman Employment**/
proc logistic data=IPV_p;
    class Age (ref='1')
          Hubage (ref='1')
          Marit_stat (ref='0')
          Hubedu (ref='0')
          V106 (ref='0')
          V714 (ref='0')
          Beat_justb (ref='0')
          D113 (ref='0')
          V024 (ref='10')
          V102 (ref='1');
    model HistIPV(event='1') = Age V106|V714 Hubedu V106|Marit_stat Hubage
Beat_justb D113 V024 V102 / clodds=pl lackfit;
    oddsratio Age;
    oddsratio V106;
      oddsratio Hubedu;
      oddsratio Hubage;
      oddsratio Marit_stat;
      oddsratio V714;
    oddsratio Beat_justb;
    oddsratio D113;
    oddsratio V024;
    oddsratio V102;
```

```
      run;

/***This interaction term is not statistically significant (p-value =0.1873 >
0.05) when WomEducation|Marital_Stat is
      already in the model

      E) Women Education X Justification of Beatings**/

proc logistic data=IPV_p;
    class Age (ref='1')
          Hubage (ref='1')
          Marit_stat (ref='0')
          Hubedu (ref='0')
          V106 (ref='0')
          V714 (ref='0')
          Beat_justb (ref='0')
          D113 (ref='0')
          V024 (ref='10')
          V102 (ref='1');
    model HistIPV(event='1') = Age V106|Beat_justb V714 Hubedu
V106|Marit_stat Hubage D113 V024 V102 / clodds=pl lackfit;
    oddsratio Age;
    oddsratio V106;
      oddsratio Hubedu;
      oddsratio Hubage;
      oddsratio Marit_stat;
      oddsratio V714;
    oddsratio Beat_justb;
    oddsratio D113;
    oddsratio V024;
    oddsratio V102;
      run;

/***This interaction term is not statistically significant (p-value =0.7989 >
0.05) when WomEducation|Marital_Stat is
      already in the model

      F) Women Education X Partner's Alcoholism**/

proc logistic data=IPV_p;
    class Age (ref='1')
          Hubage (ref='1')
          Marit_stat (ref='0')
          Hubedu (ref='0')
          V106 (ref='0')
          V714 (ref='0')
          Beat_justb (ref='0')
          D113 (ref='0')
          V024 (ref='10')
          V102 (ref='1');
    model HistIPV(event='1') = Age V106|D113 Beat_justb V714 Hubedu
V106|Marit_stat Hubage V024 V102 / clodds=pl lackfit;
    oddsratio Age;
    oddsratio V106;
      oddsratio Hubedu;
      oddsratio Hubage;
      oddsratio Marit_stat;
```

```
      oddsratio V714;
    oddsratio Beat_justb;
    oddsratio D113;
    oddsratio V024;
    oddsratio V102;
      run;

/***This interaction term is not statistically significant (p-value =0.8945 >
0.05) when WomEducation|Marital_Stat is
      already in the model

      F) Women Education X Province of Residence**/
proc logistic data=IPV_p;
    class Age (ref='1')
          Hubage (ref='1')
          Marit_stat (ref='0')
          Hubedu (ref='0')
          V106 (ref='0')
          V714 (ref='0')
          Beat_justb (ref='0')
          D113 (ref='0')
          V024 (ref='10')
          V102 (ref='1');
    model HistIPV(event='1') = Age V106|V024 D113 Beat_justb V714 Hubedu
V106|Marit_stat Hubage V102 / clodds=pl lackfit;
    oddsratio Age;
    oddsratio V106;
      oddsratio Hubedu;
      oddsratio Hubage;
      oddsratio Marit_stat;
      oddsratio V714;
    oddsratio Beat_justb;
    oddsratio D113;
    oddsratio V024;
    oddsratio V102;
      run;

/***This interaction term is not statistically significant (p-value =0.4343 >
0.05) when WomEducation|Marital_Stat is
      already in the model**/

/**Calculating Odds Ratio and CI for Interaction Terms (Marital Status and
Women Education):
      When Marit=1 and Wom Edu=1**/

data CI_Calculation;
    /* Define the coefficients and their standard errors */
    Marit_stat = -0.0285;
    V1062 = 0.0428;
    Marit_stat2_V1062 = 0.2840;
    SE_Marit_stat = 0.1366;
    SE_V1062 = 0.1040;
    SE_Marit_stat2_V1062 = 0.1543;

    /* Calculate the sum of the coefficients */
    Total_Coeff = Marit_stat + V1062 + Marit_stat2_V1062;
```

```
    /* Calculate the combined standard error */
    SE_Total_Coeff = sqrt(SE_Marit_stat**2 + SE_V1062**2 +
SE_Marit_stat2_V1062**2);

    /* Calculate the odds ratio */
    OddsRatio = exp(Total_Coeff);

    /* Calculate the confidence interval for the sum of the coefficients */
    Z = 1.96; /* Z-value for 95% CI */
    Lower_Bound = Total_Coeff - Z * SE_Total_Coeff;
    Upper_Bound = Total_Coeff + Z * SE_Total_Coeff;

    /* Exponentiate the confidence interval bounds */
    CILower = exp(Lower_Bound);
    CIUpper = exp(Upper_Bound);

    /* Keep only the result variables */
    keep Total_Coeff SE_Total_Coeff OddsRatio Lower_Bound Upper_Bound CILower
CIUpper;
run;

proc print data=CI_Calculation noobs;
run;

/*When Wom Edu=1 and  Marit=2**/
data CI_Calculation;
    /* Define the coefficients and their standard errors */
    Marit_stat = 0.4804;
    V1062 = 0.0428;
    Marit_stat2_V1062 = 0.2947;
    SE_Marit_stat = 0.1160;
    SE_V1062 = 0.1040;
    SE_Marit_stat2_V1062 = 0.1327;

    /* Calculate the sum of the coefficients */
    Total_Coeff = Marit_stat + V1062 + Marit_stat2_V1062;

    /* Calculate the combined standard error */
    SE_Total_Coeff = sqrt(SE_Marit_stat**2 + SE_V1062**2 +
SE_Marit_stat2_V1062**2);

    /* Calculate the odds ratio */
    OddsRatio = exp(Total_Coeff);

    /* Calculate the confidence interval for the sum of the coefficients */
    Z = 1.96; /* Z-value for 95% CI */
    Lower_Bound = Total_Coeff - Z * SE_Total_Coeff;
    Upper_Bound = Total_Coeff + Z * SE_Total_Coeff;

    /* Exponentiate the confidence interval bounds */
    CILower = exp(Lower_Bound);
    CIUpper = exp(Upper_Bound);

    /* Keep only the result variables */
    keep Total_Coeff SE_Total_Coeff OddsRatio Lower_Bound Upper_Bound CILower
CIUpper;
run;
```

```
proc print data=CI_Calculation noobs;
run;

/*When Wom Edu=1 and  Marit=3**
data CI_Calculation;
    /* Define the coefficients and their standard errors */
data CI_Calculation;
    Marit_stat = 0.5808;
    V1062 = 0.0428;
    Marit_stat2_V1062 = -0.0487;
    SE_Marit_stat = 0.1349;
    SE_V1062 = 0.1040;
    SE_Marit_stat2_V1062 = 0.1610;

    /* Calculate the sum of the coefficients */
    Total_Coeff = Marit_stat + V1062 + Marit_stat2_V1062;

    /* Calculate the combined standard error */
    SE_Total_Coeff = sqrt(SE_Marit_stat**2 + SE_V1062**2 +
SE_Marit_stat2_V1062**2);

    /* Calculate the odds ratio */
    OddsRatio = exp(Total_Coeff);

    /* Calculate the confidence interval for the sum of the coefficients */
    Z = 1.96; /* Z-value for 95% CI */
    Lower_Bound = Total_Coeff - Z * SE_Total_Coeff;
    Upper_Bound = Total_Coeff + Z * SE_Total_Coeff;

    /* Exponentiate the confidence interval bounds */
    CILower = exp(Lower_Bound);
    CIUpper = exp(Upper_Bound);

    /* Keep only the result variables */
    keep Total_Coeff SE_Total_Coeff OddsRatio Lower_Bound Upper_Bound CILower
CIUpper;
run;

proc print data=CI_Calculation noobs;
run;

/*When Wom Edu=2 and  Marit=1**/
data CI_Calculation;
    Marit_stat = -0.0285;
    V1062 = 0.1097;
    Marit_stat2_V1062 = 0.2287;
    SE_Marit_stat = 0.1366;
    SE_V1062 = 0.0966;
    SE_Marit_stat2_V1062 = 0.1621;

    /* Calculate the sum of the coefficients */
    Total_Coeff = Marit_stat + V1062 + Marit_stat2_V1062;

    /* Calculate the combined standard error */
    SE_Total_Coeff = sqrt(SE_Marit_stat**2 + SE_V1062**2 +
SE_Marit_stat2_V1062**2);
```

```
    /* Calculate the odds ratio */
    OddsRatio = exp(Total_Coeff);

    /* Calculate the confidence interval for the sum of the coefficients */
    Z = 1.96; /* Z-value for 95% CI */
    Lower_Bound = Total_Coeff - Z * SE_Total_Coeff;
    Upper_Bound = Total_Coeff + Z * SE_Total_Coeff;

    /* Exponentiate the confidence interval bounds */
    CILower = exp(Lower_Bound);
    CIUpper = exp(Upper_Bound);

    /* Keep only the result variables */
    keep Total_Coeff SE_Total_Coeff OddsRatio Lower_Bound Upper_Bound CILower
CIUpper;
run;

proc print data=CI_Calculation noobs;
run;

/*When Wom Edu=2 and  Marit=2**/
data CI_Calculation;
    Marit_stat = 0.4804;
    V1062 = 0.1097;
    Marit_stat2_V1062 = -0.1970;
    SE_Marit_stat = 0.1160;
    SE_V1062 =0.0966;
    SE_Marit_stat2_V1062 = 0.1314;

    /* Calculate the sum of the coefficients */
    Total_Coeff = Marit_stat + V1062 + Marit_stat2_V1062;

    /* Calculate the combined standard error */
    SE_Total_Coeff = sqrt(SE_Marit_stat**2 + SE_V1062**2 +
SE_Marit_stat2_V1062**2);

    /* Calculate the odds ratio */
    OddsRatio = exp(Total_Coeff);

    /* Calculate the confidence interval for the sum of the coefficients */
    Z = 1.96; /* Z-value for 95% CI */
    Lower_Bound = Total_Coeff - Z * SE_Total_Coeff;
    Upper_Bound = Total_Coeff + Z * SE_Total_Coeff;

    /* Exponentiate the confidence interval bounds */
    CILower = exp(Lower_Bound);
    CIUpper = exp(Upper_Bound);

    /* Keep only the result variables */
    keep Total_Coeff SE_Total_Coeff OddsRatio Lower_Bound Upper_Bound CILower
CIUpper;
run;

proc print data=CI_Calculation noobs;
run;
```

```
/*When Wom Edu=2 and  Marit=3**/
data CI_Calculation;
    Marit_stat =0.5808;
    V1062 = 0.1097;
    Marit_stat2_V1062 = -0.0441;
    SE_Marit_stat = 0.1349;
    SE_V1062 =0.0966;
    SE_Marit_stat2_V1062 = 0.1664;

    /* Calculate the sum of the coefficients */
    Total_Coeff = Marit_stat + V1062 + Marit_stat2_V1062;

    /* Calculate the combined standard error */
    SE_Total_Coeff = sqrt(SE_Marit_stat**2 + SE_V1062**2 +
SE_Marit_stat2_V1062**2);

    /* Calculate the odds ratio */
    OddsRatio = exp(Total_Coeff);

    /* Calculate the confidence interval for the sum of the coefficients */
    Z = 1.96; /* Z-value for 95% CI */
    Lower_Bound = Total_Coeff - Z * SE_Total_Coeff;
    Upper_Bound = Total_Coeff + Z * SE_Total_Coeff;

    /* Exponentiate the confidence interval bounds */
    CILower = exp(Lower_Bound);
    CIUpper = exp(Upper_Bound);

    /* Keep only the result variables */
    keep Total_Coeff SE_Total_Coeff OddsRatio Lower_Bound Upper_Bound CILower
CIUpper;
run;

proc print data=CI_Calculation noobs;
run;


/*When Wom Edu=3 and  Marit=1**/
data CI_Calculation;
    Marit_stat = -0.0285;
    V1062 = -0.4222;
    Marit_stat2_V1062 = -0.7270;
    SE_Marit_stat = 0.1366;
    SE_V1062 =0.1936;
    SE_Marit_stat2_V1062 = 0.3408;

    /* Calculate the sum of the coefficients */
    Total_Coeff = Marit_stat + V1062 + Marit_stat2_V1062;

    /* Calculate the combined standard error */
    SE_Total_Coeff = sqrt(SE_Marit_stat**2 + SE_V1062**2 +
SE_Marit_stat2_V1062**2);

    /* Calculate the odds ratio */
    OddsRatio = exp(Total_Coeff);

    /* Calculate the confidence interval for the sum of the coefficients */
```

```
    Z = 1.96; /* Z-value for 95% CI */
    Lower_Bound = Total_Coeff - Z * SE_Total_Coeff;
    Upper_Bound = Total_Coeff + Z * SE_Total_Coeff;

    /* Exponentiate the confidence interval bounds */
    CILower = exp(Lower_Bound);
    CIUpper = exp(Upper_Bound);

    /* Keep only the result variables */
    keep Total_Coeff SE_Total_Coeff OddsRatio Lower_Bound Upper_Bound CILower
CIUpper;
run;

proc print data=CI_Calculation noobs;
run;

/*When Wom Edu=3 and  Marit=2**/
data CI_Calculation;
    Marit_stat =0.4804;
    V1062 = -0.4222;
    Marit_stat2_V1062 = 0.2756;
    SE_Marit_stat = 0.1160;
    SE_V1062 =0.1936;
    SE_Marit_stat2_V1062 = 0.2822;

    /* Calculate the sum of the coefficients */
    Total_Coeff = Marit_stat + V1062 + Marit_stat2_V1062;

    /* Calculate the combined standard error */
    SE_Total_Coeff = sqrt(SE_Marit_stat**2 + SE_V1062**2 +
SE_Marit_stat2_V1062**2);

    /* Calculate the odds ratio */
    OddsRatio = exp(Total_Coeff);

    /* Calculate the confidence interval for the sum of the coefficients */
    Z = 1.96; /* Z-value for 95% CI */
    Lower_Bound = Total_Coeff - Z * SE_Total_Coeff;
    Upper_Bound = Total_Coeff + Z * SE_Total_Coeff;

    /* Exponentiate the confidence interval bounds */
    CILower = exp(Lower_Bound);
    CIUpper = exp(Upper_Bound);

    /* Keep only the result variables */
    keep Total_Coeff SE_Total_Coeff OddsRatio Lower_Bound Upper_Bound CILower
CIUpper;
run;

proc print data=CI_Calculation noobs;
run;

/*When Wom Edu=3 and  Marit=3**/
data CI_Calculation;
    Marit_stat =0.5808;
    V1062 = -0.4222;
    Marit_stat2_V1062 = 0.2751;
```

```
    SE_Marit_stat = 0.1349;
    SE_V1062 =0.1936;
    SE_Marit_stat2_V1062 = 0.3398;

    /* Calculate the sum of the coefficients */
    Total_Coeff = Marit_stat + V1062 + Marit_stat2_V1062;

    /* Calculate the combined standard error */
    SE_Total_Coeff = sqrt(SE_Marit_stat**2 + SE_V1062**2 +
SE_Marit_stat2_V1062**2);

    /* Calculate the odds ratio */
    OddsRatio = exp(Total_Coeff);

    /* Calculate the confidence interval for the sum of the coefficients */
    Z = 1.96; /* Z-value for 95% CI */
    Lower_Bound = Total_Coeff - Z * SE_Total_Coeff;
    Upper_Bound = Total_Coeff + Z * SE_Total_Coeff;

    /* Exponentiate the confidence interval bounds */
    CILower = exp(Lower_Bound);
    CIUpper = exp(Upper_Bound);

    /* Keep only the result variables */
    keep Total_Coeff SE_Total_Coeff OddsRatio Lower_Bound Upper_Bound CILower
CIUpper;
run;

proc print data=CI_Calculation noobs;
run;

/**Find the absolute prevalence of the IPV relative to the overall
representative provincial population for each province**/
proc freq data=IPV_p;
    tables V024*HistIPV / nocum;
    where HistIPV = 1; /* Include only women who experienced IPV */
    title 'Count of Women Who Experienced IPV by Province of Residence';
run;

/***Creating permanent dataset**/
data IPV3.IPV_mod1;
set IPV_p;
run;
```
